# Supplementary material for: Effects of an anti-lipogenic low-carbohydrate high polyunsaturated fat diet or a healthy Nordic diet versus usual care on liver fat and cardiometabolic disorders in type 2 diabetes or prediabetes: a randomized controlled trial (NAFLDiet)
Source: Nat Commun. 2025 Dec 13;16:11130. doi: 10.1038/s41467-025-65613-2 (PMC12705689; doi:10.1038/s41467-025-65613-2)
Supplement: Supplementary file 1 — Supplementary Information [file 41467_2025_65613_MOESM1_ESM.pdf]

Effects of an anti-lipogenic low-carbohydrate high polyunsaturated fat diet or a healthy Nordic diet versus usual care on liver fat and cardiometabolic disorders in type 2 diabetes or prediabetes: a randomized controlled trial (NAFLDiet)

Supplementary Material

Fridén M[1], Rosqvist F[1,2], Kullberg J[3,4], Berglund L[1,5,6], Vessby J[7,] Martinell M[1], Carlsson P-O[8], Hulthe J[4], Johansson L[4], Ahmad N[3], Johansson H-E[1], Rorsman F[7], Sundström J[7], Lind L[7], Landberg R[9,10], Orho-Melander M[11,] Ahlström H[3], Risérus U[1*]

[1]Department of Public Health and Caring Sciences, Uppsala University, Uppsala, Sweden.

[2]Department of Food Studies, Nutrition and Dietetics, Uppsala University, Uppsala, Sweden.

[3]Department of Surgical Sciences, Radiology, Uppsala University, Uppsala, Sweden.

[4]Antaros Medical AB, Gothenburg, Sweden.

[5]School of Health and Welfare, Dalarna University, Falun, Sweden.

[6]Epistat AB, Uppsala, Sweden.

[7]Department of Medical Sciences, Uppsala University, Uppsala, Sweden.

[8]Department of Medical Cell Biology, Uppsala University, Uppsala, Sweden.

[9]Division of Food and Nutrition Science, Department of Life Sciences, Chalmers University of Technology, Gothenburg, Sweden.

[10]Wallenberg Laboratory and Department of Molecular and Clinical Medicine, Institute of Medicine, Sahlgrenska Academy, University of Gothenburg, Gothenburg, Sweden.

[11]Department of Clinical Sciences Malmö, Lund University, Lund, Sweden.

*Corresponding author

Ulf Risérus (ulf.riserus@uu.se)

Table of contents

Protocol figure……………………………………………………………………………………….3

Deviations from the original statistical analysis plan (SAP)……………………………………...4

Information on the diets…………………………………………………………………………...5-6

Missing data………………………………………………………………………………………7

Evaluation of the diets…………………………………………………………………………….8

Biomarker analyses of alkylresorcinols…………….……………………………………………9

Per-protocol analyses……………….……………………………………………………………10

Subgroup analyses……………………………………………………………..………..11-16

Adverse events…………………………………………………………………………………..17

Co-interventions………………………………………………………………………………...18-20

Descriptive data on remission rates of prediabetes and NAFLD………………………………..21

PNPLA3 genotype distributions among NAFLD subgroups……………………………………22

Causal mediation analysis………………………………………………………………………23-25

References……………………………………………………………………………………….26

Supplementary Note 1

Supplementary Note 2

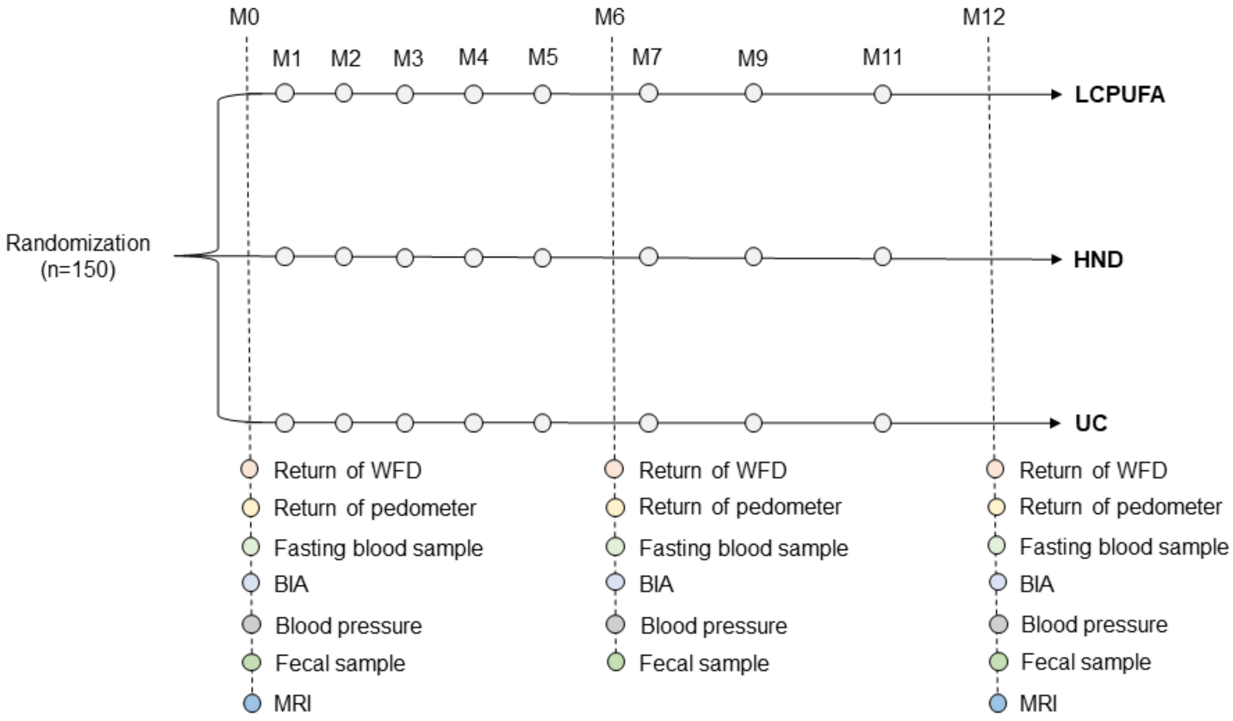

Supplementary Figure 1. Protocol figure. On each monthly or bimonthly meeting (M0-M12), participants received key food items in a bag. BIA, Bioelectrical Impedance Analysis; HND, Healthy Nordic Diet; LCPUFA, Lower Carbohydrate Polyunsaturated Fatty Acids; MRI, Magnetic Resonance Imaging; UC, Usual Care; WFD, Weighed Food Diaries.

Deviations from the original statistical analysis plan (SAP)

1. Addition of FIB-4 as a secondary outcome. Added to ClinicalTrials.gov the 23rd of July 2022, but was not added to the original SAP (which was uploaded 12th of May 2021).

2. Addition of ALAT, ASAT and GGT as exploratory outcomes were added to the manuscript after completion of the study, but not to the SAP nor ClinicalTrials.gov.

3. Addition of dietary and biomarker variables (i.e., plasma alkylresorcinols and fatty acids) relevant for the evaluation of adherence as a secondary outcome. These outcomes were neither added to ClinicalTrials.gov nor to the original SAP.

4. The estimation of the ITT-effect was decided to apply for all primary and secondary outcomes, not just for HbA1c, LDL cholesterol, triglycerides and systolic- and diastolic blood pressure, as prespecified on page 12 in the original SAP. The main reason for this decision was that the ITT-effect was our primary effect of interest (prespecified in the original SAP), and hence should apply to all outcomes of interest. As the ITT-effect is an unbiased effect of treatment assignment at baseline, whereas the per-protocol effect may be biased in case of patient dropouts and non-adherence, the ITT-effect for all outcomes was deemed more reasonable.

5. Information on number of steps from pedometers are not presented in the main text due to logistic difficulties in providing participants with a fully functional one at each time point (i.e. some pedometers stopped working or were lost over the follow-up period).

6. Subgroup analyses based on compliers will not be conducted. The ITT-effect cannot be estimated if compliance to each diet is non-random and affected by pre- and post-baseline variables. In addition, compliance was not prespecified on an individual basis, hence the difficulties in estimating the non-naïve per-protocol effect.

7. Sensitivity analysis whereby weight change is included as a covariate in the model has been extended to form a causal mediation analysis to estimate the proportion mediated by weight change of the primary outcome (referred to as a post-hoc analysis in the study) (see causal mediation analysis section on page 23-25). The reason for the extension is that traditional methods such as including the mediator as a covariate in the regression model does not usually consider mediator-outcome confounders and interactions between the exposure and the mediator, potentially leading to biased estimates of the direct and indirect effects.

8. Additional sensitivity analyses not prespecified in the SAP have been performed (see statistical analysis section of the manuscript for detailed information).

9. Other secondary and exploratory outcomes and analyses will be incorporated in later publications.

Supplementary Table 1. Dietary goals and key food items provided for each of the three diets.

	LCPUFA	HND	UC
Dietary goal 1	Eat at least a handful (40 g) of nuts each day (emphasized)	Eat at least 2 portions of whole-grain (of rye, oats, barley) each day (emphasized)	Eat at least 600 grams of fruits, vegetables and berries each day (emphasized)
Dietary goal 2	Eat at least 2 tbsps. of seeds each day (emphasized)	Eat at least 1 slice of whole-grain bread (of rye, oats, barley) each day (emphasized)	Replace refined grains with whole-grains
Dietary goal 3	Eat at least 2 tbsps. of sunflower oil each day (emphasized)	Limit the amount of fat (emphasized)	Eat less salt
Dietary goal 4	Limit the amount of potatoes, rice, pasta, bread and other cereals (emphasized)	Put focus on food items emphasized under each dietary goal* (emphasized)	Eat more nuts and seeds
Dietary goal 5	Limit the amount of desserts, candy, crisps, sugar-sweetened beverages and juice	Limit the amount of desserts, candy, crisps, sugar-sweetened beverages and juice	Eat less sugar
Dietary goal 6	Avoid butter and replace whole-fat dairy with low-fat dairy	Avoid butter and replace whole-fat dairy with low-fat dairy	Replace whole-fat dairy with low-fat dairy
Dietary goal 7	Use liquid margarine/vegetable oils when cooking and vegetable oil-based bread spread on bread	Use liquid margarine/vegetable oils when cooking and vegetable oil-based bread spread on bread, although in small quantities	Replace unhealthy fats with healthy fats
Dietary goal 8	Replace some of the red meat with fish, poultry or vegetable protein sources	Replace some of the red meat with fish, poultry or vegetable protein sources	Eat less red and processed red meat

Dietary goal 9	Eat at least 2 portions of fatty fish each week	Eat at least 2 portions of fatty fish each week	Eat fish and seafood 2-3 times each week
Dietary goal 10	Increase the amount of fruits, vegetables and berries	Increase the amount of Swedish fruits, vegetables and berries	Drink less sugar-sweetened beverages
Key food items provided	Walnuts, sunflower seeds, pumpkin seeds, cashew nuts and sunflower oil	Whole-grain muesli, crisp bread, frozen raspberries, oats, oat bran, beans, lentils, oat rice, low-fat margarine, almonds	Carrots, whole-grain muesli, crisp bread, frozen peas and frozen mango

*Whole-grains include rye, barley and oats but not wheat; Bread includes rye, barley and oats but not wheat; Vegetable fat source is rapeseed oil or margarine with a mix of rapeseed-, sunflower seed- and linseed oil; Fish include salmon, mackerel and herring; Fruits include apples, pears and plums; Berries include blueberries, raspberries, lingonberries, hawthorn, strawberries, wild strawberries, cloudberries and black- and redcurrant. HND, Healthy Nordic Diet; LCPUFA, Low Carbohydrate Polyunsaturated Fatty Acids; UC, Usual Care.

Supplementary Table 2. Missing data on primary, secondary and exploratory outcomes[1].

	LCPUFA M12-M0 (n=54)	HND M12-M0 (=51)	UC M12-M0 (n=43)	Total (n=148)
Dietary variables	4 (7)	3 (6)	3 (7)	10 (7)
Plasma PL FA	5 (9)	1 (2)	3 (7)	9 (6)
Plasma TAG FA	6 (11)	1 (2)	3 (7)	10 (7)
Alkylresorcinols	11 (20)	5 (10)	4 (9)	20 (14)
Liver fat	9 (17)	5 (10)	7 (16)	21 (14)
Weight	4 (7)	1 (2)	3 (7)	8 (5)
Fasting glucose	5 (9)	1 (2)	3 (7)	9 (6)
HbA1c	5 (9)	1 (2)	3 (7)	9 (6)
Total cholesterol	5 (9)	1 (2)	3 (7)	9 (6)
HOMA-IR	5 (9)	1 (2)	3 (7)	9 (6)
LDL cholesterol	5 (9)	1 (2)	3 (7)	9 (6)
HDL cholesterol	5 (9)	1 (2)	3 (7)	9 (6)
Triglycerides	5 (9)	1 (2)	3 (7)	9 (6)
ApoB	6 (11)	2 (4)	3 (7)	11 (7)
ApoA1	6 (11)	2 (4)	3 (7)	11 (7)
Insulin	5 (9)	1 (2)	3 (7)	9 (6)
FIB-4	5 (9)	1 (2)	4 (9)	10 (7)
SBP	5 (9)	1 (2)	3 (7)	9 (6)
DBP	5 (9)	1 (2)	3 (7)	9 (6)
CRP	5 (9)	1 (2)	3 (7)	9 (6)
ALAT	5 (9)	1 (2)	3 (7)	9 (6)
ASAT	5 (9)	1 (2)	3 (7)	9 (6)
GGT	5 (9)	1 (2)	3 (7)	9 (6)

[1]Data are presented as n (%).
ALAT, Alanine Aminotransferase; ASAT, Aspartate Aminotransferase; ApoA1, Apolipoprotein A1; ApoB, Apolipoprotein B; CRP, C-Reactive Protein; DBP, Diastolic Blood Pressure; EMM, Estimated Marginal Means; FA, Fatty Acids; FIB-4, Fibrosis-4; GGT, Gamma Glutamyltransferase; HbA1c, Hemoglobin A1c; HDL, High-Density Lipoprotein; HND, Healthy Nordic Diet; LCPUFA, Low Carbohydrate Polyunsaturated Fatty Acids; LDL, Low-Density Lipoprotein; M0, Baseline; M12, Month 12; PL, Phospholipids; SBP, Systolic Blood Pressure; TAG, Triacylglycerols; UC, Usual Care.

Supplementary Table 3. Evaluation of the diets[1].

	LCPUFA (n=50)	HND (n=50)	UC (n=40)
Satisfaction with diet	1.5 (1.0)	1.0 (1.0)	1.0 (1.0)
Motivation to continue with diet	2.0 (1.0)	2.0 (1.0)	1.5 (1.0)
Economic cost of diet	3.0 (2.0)	4.0 (1.0)	4.0 (1.0)
Time spent planning around diet	3.0 (1.0)	4.0 (1.0)	3.0 (1.0)
Difficulties adhering to diet away	3.0 (2.0)	2.0 (1.0)	3.0 (2.0)
Feeling of everyday energy	3.0 (1.0)	2.0 (1.0)	2.0 (1.0)
Feeling of hunger	4.0 (1.0)	4.0 (2.0)	4.0 (1.0)
Feeling of healthfulness	2.0 (1.0)	1.0 (1.0)	2.0 (1.0)

[1]Data are presented as medians (interquartile range (IQR)).
Each statement is followed by five possible answers to choose from (1=fully agree, 2=agree, 3=neither agree nor disagree, 4=do not agree, 5=do not agree at all). Statement 1-8 are presented below.
Satisfaction with diet: I am overall satisfied with the diet that I was assigned.
Motivation to continue with diet: I am feeling motivated to continue this diet in the future.
Economic cost of diet: I feel like this diet is expensive.
Time spent planning around diet: I feel like I am spending much time planning what to eat every day.
Difficulties adhering to diet away: I feel like this particular diet is difficult to adhere to on restaurants or when eating at others.
Feeling of everyday energy: I feel like this diet gives me much energy.
Feeling of hunger: I often feel hungry on this diet.
Feeling of healthfulness: I feel like this diet contributes to a healthier lifestyle.
LCPUFA, Low Carbohydrate Polyunsaturated Fatty Acids; HND, Healthy Nordic Diet; UC, Usual Care.

Supplementary Table 4. Within-group changes and overall test of the difference in means between groups of biomarkers of dietary intake of whole-grain[1].

	LCPUFA (M0)[1]	HND (M0)[1]	UC (M0)[1]	EMM (95% CI)[2] M12-M0 LCPUFA (n=54)	EMM (95% CI)[2] M12-M0 HND (n=51)	EMM (95% CI)[2] M12-M0 UC (n=43)	P-value[3]
Total AR	44.4 (42.8)	54.2 (72.6)	51.1 (68.9)	-25.51 (-38.71, -12.32)	-9.38 (-22.89, 4.14)	-16.08 (-27.61, -4.54)	0.13
17:0/21:0	0.2 ± 0.1	0.2 ± 0.1	0.2 ± 0.1	0.05 (0.01, 0.09)	0.07 (0.04, 0.11)	0.03 (-0.01, 0.07)	0.35

[1]Data are presented as mean ± standard deviation (SD) or median (interquartile range (IQR)) for descriptive values at baseline (M0) (n=148) for each diet.
[2]Estimated marginal means or medians (EMM) with corresponding 95% CI are presented for the change in alkylresorcinols within each diet group. EMMs are conditioned on baseline value of the outcome, presence of type 2 diabetes and sex.
[3]Two-sided p-values are calculated from the general linear model (GLM) or corresponding Kruskal-Wallis (KW) test for the overall test of between-group differences.
AR, Alkylresorcinols; EMM, Estimated Marginal Means; HND, Healthy Nordic Diet; LCPUFA, Low Carbohydrate Polyunsaturated Fatty Acids; M0, Baseline; M12, Month 12; UC, Usual Care.

Supplementary Table 5. Between-group differences of primary and secondary outcomes for per-protocol effect estimates[1].

	LCPUFA vs HND	P-value	LCPUFA vs UC	P-value	HND vs UC	P-value
Liver fat (%)	0.53 (-0.21, 1.25)	0.19	-1.40 (-2.33, -0.35)	0.01	-2.00 (-3.19, -0.84)	<0.001
Weight (kg)	2.53 (0.80, 4.26)	0.004	-0.34 (-2.16, 1.49)	0.72	-2.87 (-4.72, -1.01)	0.003
HbA1c (mmol/mol)	1.77 (0.34, 3.19)	0.02	-0.40 (-1.93, 1.13)	0.60	-2.17 (-3.70, -0.64)	0.01
Total cholesterol (mmol/L)	0.05 (-0.22, 0.33)	0.71	-0.32 (-0.62, -0.02)	0.03	-0.37 (-0.66, -0.08)	0.01
LDL cholesterol (mmol/L)	0.03 (-0.20, 0.25)	0.82	-0.28 (-0.51, -0.04)	0.02	-0.30 (-0.54, -0.07)	0.01
Triglycerides (mmol/L)	0.11 (-0.13, 0.36)	0.36	-0.25 (-0.51, 0.004)	0.05	-0.37 (-0.63, -0.11)	0.01
CRP (mg/L)	0.25 (-0.10, 0.65)	0.14	-0.20 (-0.66, 0.19)	0.34	-0.49 (-0.84, -0.15)	0.01

[1]Per-protocol effect estimates with corresponding 95% confidence intervals (CI) for primary and secondary outcomes.

Estimates and two-sided p-values are derived from general linear models (GLMs) or the Hodges Lehman estimator to retrieve median differences and 95% CI.

CRP, C-Reactive Protein; HbA1c, Hemoglobin A1C; HND, Healthy Nordic Diet; LCPUFA, Low Carbohydrate Polyunsaturated Fatty Acids; LDL, Low-Density Lipoprotein; UC, Usual Care.

For liver fat, n (LCPUFA) = 45, n (HND) = 46, n (UC) = 36. For weight, n (LCPUFA) = 50, n (HND) = 50, n (UC) = 40. For all other outcomes, n (LCPUFA) = 49, n (HND) = 50, n (UC) = 40.

Supplementary Table 6. Within-group changes and overall test of the difference in means/medians between groups of primary and secondary outcomes for subgroups[1].

	LCPUFA (M0)[1]	HND (M0)[1]	UC (M0)[1]	EMM (95% CI)[2] M12-M0 LCPUFA (n=54)	EMM (95% CI)[2] M12-M0 HND (n=51)	EMM (95% CI)[2] M12-M0 UC (n=43)	P-value[3]
Stratified by sex							
Men				n=32	n=31	n=27	
Liver fat (%)	6.3 (7.3)	6.5 (9.8)	8.7 (10.4)	-0.67 (-1.18, -0.15)	-1.23 (-2.08, -0.39)	0.52 (-0.32, 1.36)	0.09
HbA1c (mmol/L)	43.0 (15.0)	39.5 (10.0)	39.0 (13.0)	0.62 (-0.79, 2.03)	-1.66 (-3.07, -0.25)	0.80 (-0.73, 2.34)	0.03
Total cholesterol (mmol/L)	4.4 ± 1.2	4.8 ± 1.2	5.0 ± 1.1	-0.42 (-0.66, -0.19)	-0.44 (-0.68, -0.21)	-0.08 (-0.34, 0.17)	0.08
LDL cholesterol (mmol/L)	2.7 ± 1.0	3.0 ± 1.0	3.1 ± 1.0	-0.31 (-0.50, -0.12)	-0.34 (-0.53, -0.15)	-0.07 (-0.27, 0.14)	0.12
HDL cholesterol (mmol/L)	1.2 ± 0.2	1.2 ± 0.3	1.2 ± 0.2	-0.01 (-0.07, 0.05)	0.06 (0.00, 0.13)	0.04 (-0.03, 0.11)	0.29
Triglycerides (mmol/L)	1.4 (0.7)	1.3 (1.0)	1.4 (0.8)	-0.21 (-0.45, 0.02)	-0.42 (-0.65, -0.18)	0.02 (-0.23, 0.27)	0.04
ApoB (g/L)	0.9 ± 0.3	0.9 ± 0.3	0.9 ± 0.3	-0.07 (-0.13, -0.02)	-0.09 (-0.14, -0.03)	-0.04 (-0.10, 0.02)	0.49
ApoA1 (g/L)	1.4 ± 0.2	1.5 ± 0.2	1.4 ± 0.2	-0.05 (-0.10, 0.00)	-0.02 (-0.07, 0.03)	-0.01 (-0.06, 0.05)	0.51
Women				n=22	n=20	n=16	
Liver fat (%)	6.1 (6.4)	6.3 (2.9)	8.6 (12.1)	-0.55 (-0.99, -0.11)	-0.57 (-1.42, 0.28)	2.16 (-0.05, 4.37)	0.06
HbA1c (mmol/L)	41.0 (9.0)	40.0 (10.0)	40.5 (4.0)	-0.08 (-1.44, 1.28)	-0.76 (-2.12, 0.60)	0.49 (-1.16, 2.15)	0.51
Total cholesterol (mmol/L)	5.0 ± 1.3	5.4 ± 1.2	5.0 ± 1.2	-0.28 (-0.61, 0.06)	-0.26 (-0.57, 0.06)	0.08 (-0.31, 0.47)	0.31
LDL cholesterol (mmol/L)	3.0 ± 1.2	3.4 ± 1.1	3.2 ± 1.2	-0.31 (-0.59, -0.03)	-0.27 (-0.53, 0.00)	0.07 (-0.25, 0.39)	0.16

HDL cholesterol (mmol/L)	1.6 ± 0.4	1.5 ± 0.3	1.4 ± 0.3	0.07 (-0.01, 0.15)	0.03 (-0.05, 0.10)	0.03 (-0.06, 0.12)	0.71
Triglycerides (mmol/L)	1.2 (0.7)	1.5 (0.8)	1.3 (0.7)	-0.10 (-0.32, 0.13)	-0.15 (-0.37, 0.08)	0.16 (-0.11, 0.43)	0.20
ApoB (g/L)	0.9 ± 0.3	1.0 ± 0.3	1.0 ± 0.3	-0.10 (-0.18, -0.02)	-0.04 (-0.11, 0.03)	0.00 (-0.09, 0.09)	0.22
ApoA1 (g/L)	1.6 ± 0.3	1.7 ± 0.2	1.6 ± 0.2	0.04 (-0.03, 0.10)	-0.03 (-0.09, 0.03)	-0.06 (-0.13, 0.01)	0.12
Stratified by T2D diagnosis							
T2D				n=30	n=23	n=28	
Liver fat (%)	7.3 (6.4)	7.5 (8.7)	8.9 (10.4)	-0.85 (-1.36, -0.34)	-1.65 (-2.47, -0.83)	1.58 (0.45, 2.71)	0.0002
HbA1c (mmol/L)	48.0 (10.0)	47.0 (11.0)	44.0 (11.0)	-0.05 (-1.72, 1.61)	-2.82 (-4.69, -0.95)	1.14 (-0.55, 2.83)	0.01
Total cholesterol (mmol/L)	4.0 ± 1.1	4.7 ± 1.2	4.6 ± 1.0	-0.19 (-0.50, 0.11)	-0.27 (-0.60, 0.07)	0.19 (-0.11, 0.49)	0.07
LDL cholesterol (mmol/L)	2.3 ± 0.9	2.9 ± 1.0	2.8 ± 1.0	-0.14 (-0.38, 0.10)	-0.28 (-0.54, -0.02)	0.13 (-0.09, 0.36)	0.04
HDL cholesterol (mmol/L)	1.3 ± 0.4	1.3 ± 0.3	1.3 ± 0.2	0.08 (0.02, 0.15)	0.10 (0.02, 0.18)	0.08 (0.00, 0.15)	0.91
Triglycerides (mmol/L)	1.4 (0.8)	1.4 (0.9)	1.5 (0.8)	-0.15 (-0.46, 0.15)	-0.40 (-0.71, -0.10)	0.18 (-0.10, 0.46)	0.02
ApoB (g/L)	0.8 ± 0.2	0.9 ± 0.2	0.9 ± 0.2	-0.07 (-0.14, 0.00)	-0.06 (-0.13, 0.01)	0.03 (-0.03, 0.09)	0.04
ApoA1 (g/L)	1.5 ± 0.3	1.5 ± 0.2	1.5 ± 0.2	0.03 (-0.02, 0.09)	0.02 (-0.05, 0.08)	-0.01 (-0.06, 0.05)	0.60
Prediabetes				n=24	n=28	n=15	
Liver fat (%)	4.1 (7.3)	6.3 (6.1)	7.2 (10.1)	-0.37 (-1.32, 0.57)	-0.50 (-0.98, -0.01)	-0.31 (-2.18, 1.55)	0.75
HbA1c (mmol/L)	37.0 (5.0)	37.0 (4.0)	38.0 (4.0)	0.73 (-0.17, 1.64)	-0.02 (-0.82, 0.79)	-0.20 (-1.35, 0.95)	0.33
Total cholesterol (mmol/L)	5.4 ± 1.0	5.4 ±1.2	5.8 ± 0.8	-0.46 (-0.69, -0.23)	-0.45 (-0.66, -0.25)	-0.27 (-0.57, 0.03)	0.53
LDL cholesterol (mmol/L)	3.6 ± 0.8	3.5 ± 1.2	3.9 ± 0.8	-0.42 (-0.62, -0.22)	-0.37 (-0.55, -0.19)	-0.22 (-0.48, 0.04)	0.47
HDL cholesterol (mmol/L)	1.4 ± 0.3	1.4 ± 0.3	1.4 ± 0.3	-0.02 (-0.09, 0.04)	0.00 (-0.05, 0.06)	0.03 (-0.05, 0.11)	0.58

Triglycerides (mmol/L)	1.3 (0.5)	1.3 (0.8)	1.3 (0.8)	-0.21 (-0.41, -0.02)	-0.19 (-0.36, -0.02)	-0.09 (-0.33, 0.15)	0.72
ApoB (g/L)	1.0 ± 0.2	1.0 ± 0.3	1.1 ± 0.2	-0.08 (-0.14, -0.02)	-0.08 (-0.14, -0.03)	-0.10 (-0.18, -0.02)	0.93
ApoA1 (g/L)	1.5 ± 0.2	1.6 ± 0.2	1.5 ± 0.2	-0.06 (-0.12, 0.00)	-0.05 (-0.11, 0.00)	-0.03 (-0.10, 0.05)	0.79
Stratified by NAFLD status							
NAFLD				n=29	n=33	n=30	
Liver fat (%)	10.2 (6.0)	8.6 (7.7)	13.2 (9.6)	-0.01 (-1.66, 1.64)	-1.15 (-2.44, 0.14)	1.63 (-0.06, 3.33)	0.11
HbA1c (mmol/L)	44.5 (13.0)	40.0 (13.0)	44.0 (12.0)	0.97 (-0.60, 2.55)	-1.87 (-3.33, -0.41)	1.03 (-0.55, 2.61)	0.01
Total cholesterol (mmol/L)	4.5 ± 1.3	5.3 ± 1.3	5.0 ± 1.1	-0.13 (-0.43, 0.17)	-0.38 (-0.64, -0.12)	0.01 (-0.28, 0.30)	0.14
LDL cholesterol (mmol/L)	2.8 ± 1.1	3.4 ± 1.2	3.1 ± 1.0	-0.13 (-0.39, 0.12)	-0.33 (-0.54, -0.11)	-0.03 (-0.27, 0.21)	0.17
HDL cholesterol (mmol/L)	1.2 ± 0.2	1.3 ± 0.3	1.2 ± 0.2	0.08 (0.01, 0.14)	0.05 (0.00, 0.11)	0.04 (-0.03, 0.10)	0.66
Triglycerides (mmol/L)	1.5 (0.6)	1.6 (0.8)	1.6 (0.7)	-0.22 (-0.49, 0.05)	-0.33 (-0.56, -0.09)	0.16 (-0.10, 0.42)	0.02
ApoB (g/L)	0.9 ± 0.3	1.0 ± 0.3	1.0 ± 0.2	-0.05 (-0.12, 0.03)	-0.07 (-0.13, 0.00)	-0.02 (-0.09, 0.05)	0.61
ApoA1 (g/L)	1.5 ± 0.2	1.5 ± 0.2	1.5 ± 0.2	0.03 (-0.02, 0.09)	0.00 (-0.05, 0.04)	-0.03 (-0.08, 0.03)	0.29
No NAFLD				n=25	n=18	n=13	
Liver fat (%)	3.3 (1.4)	3.6 (1.9)	3.7 (1.5)	-0.65 (-0.85, -0.45)	-0.73 (-1.41, -0.05)	0.34 (-0.24, 0.91)	0.004
HbA1c (mmol/L)	40.0 (11.0)	39.5 (7.0)	38.0 (5.0)	0.26 (-0.88, 1.41)	-0.32 (-1.65, 1.01)	-0.18 (-1.80, 1.44)	0.78
Total cholesterol (mmol/L)	4.7 ± 1.2	4.7 ± 1.0	5.0 ± 1.1	-0.46 (-0.71, -0.21)	-0.34 (-0.63, -0.06)	0.01 (-0.33, 0.35)	0.07
LDL cholesterol (mmol/L)	2.8 ± 1.1	2.9 ± 0.9	3.1 ± 1.2	-0.41 (-0.59, -0.23)	-0.31 (-0.53, -0.10)	0.05 (-0.20, 0.30)	0.01
HDL cholesterol (mmol/L)	1.5 ± 0.5	1.4 ± 0.3	1.4 ± 0.3	-0.02 (-0.11, 0.06)	0.05 (-0.05, 0.15)	0.07 (-0.04, 0.18)	0.29
Triglycerides (mmol/L)	1.3 (0.5)	1.1 (0.4)	1.0 (0.7)	-0.05 (-0.24, 0.14)	-0.25 (-0.46, -0.03)	-0.21 (-0.46, 0.05)	0.32

ApoB (g/L)	0.9 ± 0.2	0.9 ± 0.2	0.9 ± 0.3	-0.10 (-0.15, -0.04)	-0.08 (-0.14, -0.01)	-0.02 (-0.10, 0.05)	0.26
ApoA1 (g/L)	1.6 ± 0.3	1.6 ± 0.2	1.6 ± 0.2	-0.06 (-0.13, 0.00)	-0.03 (-0.10, 0.05)	0.00 (-0.08, 0.09)	0.43
Stratified by PNPLA3 (I148M) genotype							
PNPLA3 (CC)				n=33	n=32	n=22	
Liver fat (%)	4.9 (6.6)	6.4 (5.4)	8.3 (10.8)	-0.80 (-1.13, ´-0.47)	-1.28 (-2.05, -0.51)	1.07 (-0.80, 2.95)	0.003
HbA1c (mmol/L)	44.0 (12.0)	41.0 (11.0)	39.0 (12.0)	0.41 (-0.83, 1.65)	-1.74 (-2.99, -0.49)	2.00 (0.42, 3.58)	0.001
Total cholesterol (mmol/L)	4.8 ± 1.4	5.2 ± 1.3	5.2 ± 1.1	-0.28 (-0.55, -0.01)	-0.49 (-0.75, -0.23)	0.14 (-0.19, 0.46)	0.01
LDL cholesterol (mmol/L)	2.9 ± 1.2	3.3 ± 1.1	3.4 ± 1.0	-0.23 (-0.46, 0.00)	-0.40 (-0.61, -0.20)	0.14 (-0.13, 0.40)	0.004
HDL cholesterol (mmol/L)	1.4 ± 0.4	1.4 ± 0.3	1.2 ± 0.3	0.06 (-0.01, 0.12)	0.03 (-0.03, 0.10)	0.04 (-0.04, 0.12)	0.83
Triglycerides (mmol/L)	1.3 (0.6)	1.4 (1.0)	1.5 (0.6)	-0.30 (-0.49, -0.11)	-0.36 (-0.56, -0.17)	0.17 (-0.06, 0.41)	0.001
ApoB (g/L)	0.9 ± 0.3	1.0 ± 0.3	1.0 ± 0.2	-0.06 (-0.12, -0.01)	-0.10 (-0.15, -0.04)	0.01 (-0.06, 0.08)	0.046
ApoA1 (g/L)	1.6 ± 0.3	1.6 ± 0.2	1.5 ± 0.2	0.01 (-0.05, 0.06)	-0.02 (-0.08, 0.03)	-0.02 (-0.08, 0.05)	0.70
PNPLA3 (CG/GG)				n=21	n=19	n=21	
Liver fat (%)	6.6 (7.1)	6.5 (9.4)	8.9 (10.9)	-0.12 (-1.23, 0.99)	-0.39 (-1.50, 0.71)	0.78 (-0.62, 2.18)	0.59
HbA1c (mmol/L)	40.0 (13.0)	38.0 (4.0)	41.0 (7.0)	0.74 (-0.92, 2.40)	-0.99 (-2.65, 0.68)	-0.19 (-1.82, 1.43)	0.33
Total cholesterol (mmol/L)	4.4 ± 1.0	4.8 ± 1.2	4.8 ± 1.1	-0.44 (-0.71, -0.17)	-0.09 (-0.35, 0.17)	-0.09 (-0.36, 0.19)	0.09
LDL cholesterol (mmol/L)	2.7 ± 0.9	3.1 ± 1.1	2.9 ± 1.1	-0.38 (-0.63, -0.13)	-0.12 (-0.34, 0.11)	-0.15 (-0.38, 0.08)	0.24
HDL cholesterol (mmol/L)	1.3 ± 0.3	1.3 ± 0.3	1.4 ± 0.2	-0.05 (-0.12, 0.03)	0.08 (0.01, 0.14)	0.06 (-0.02, 0.13)	0.02
Triglycerides (mmol/L)	1.4 (0.7)	1.3 (0.8)	1.2 (0.8)	-0.08 (-0.42, 0.26)	-0.15 (-0.46, 0.16)	0.04 (-0.27, 0.35)	0.66
ApoB (g/L)	0.9 ± 0.2	0.9 ± 0.3	0.9 ± 0.3	-0.09 (-0.16, -0.01)	-0.02 (-0.09, 0.05)	-0.06 (-0.13, 0.01)	0.37
ApoA1 (g/L)	1.4 ± 0.2	1.5 ± 0.2	1.5 ± 0.2	-0.05 (-0.13, 0.03)	-0.01 (-0.08, 0.06)	-0.01 (-0.07, 0.06)	0.67

[1]Data are presented as mean ± standard deviation (SD) or median (interquartile range (IQR)) for descriptive values at baseline (M0) (n=142 for liver fat and n=148 for all other) for each diet.

[2]Estimated marginal means or medians (EMM) with corresponding 95% confidence intervals (CI) are presented for the change in primary and secondary outcomes within each diet group. EMMs are conditioned on baseline value of the outcome, presence of type 2 diabetes and sex.

[3]Two-sided p-values are calculated from the general linear model (GLM) or corresponding Kruskal-Wallis (KW) test for the overall test of between-group differences.

ApoA1, Apolipoprotein A1; ApoB, Apolipoprotein B; CRP, C-Reactive Protein; DBP, Diastolic Blood Pressure; EMM, Estimated Marginal Means; FIB-4, Fibrosis-4; HbA1c, Hemoglobin A1c; HDL, High-Density Lipoprotein; HND, Healthy Nordic Diet; LCPUFA, Low Carbohydrate Polyunsaturated Fatty Acids; LDL, Low-Density Lipoprotein; M0, Baseline; M12, Month 12; SBP, Systolic Blood Pressure; UC, Usual Care.

Supplementary Table 7. Between-group differences of primary and secondary outcomes for subgroups[1].

	LCPUFA vs HND	P-value	LCPUFA vs UC	P-value	HND vs UC	P-value
Stratified by sex						
Men						
HbA1c (mmol/L)	2.28 (0.27, 4.30)	0.03	-0.18 (-2.27, 1.90)	0.86	-2.47 (-4.55, -0.38)	0.02
Triglycerides (mmol/L)	0.21 (-0.13, 0.54)	0.23	-0.23 (-0.58, 0.11)	0.19	-0.44 (-0.78, -0.09)	0.01
Stratified by T2D diagnosis						
T2D						
Liver fat (%)	0.80 (-0.15, 1.76)	0.10	-2.43 (-3.66, -1.20)	0.0001	-3.23 (-4.61, -1.85)	<0.0001
HbA1c (mmol/L)	2.77 (0.24, 5.29)	0.03	-1.19 (-3.57, 1.19)	0.32	-3.96 (-6.46, -1.46)	0.002
LDL cholesterol (mmol/L)	0.13 (-0.23, 0.50)	0.47	-0.28 (-0.61, 0.06)	0.11	-0.41 (-0.75, -0.07)	0.02
Triglycerides (mmol/L)	0.25 (-0.18, 0.69)	0.25	-0.33 (-0.76, 0.10)	0.13	-0.58 (-1.00, -0.17)	0.01
ApoB (g/L)	-0.01 (-0.11, 0.09)	0.90	-0.10 (-0.20, -0.01)	0.03	-0.10 (-0.19, 0.00)	0.04
Stratified by NAFLD status						
NAFLD						
HbA1c (mmol/L)	2.85 (0.69, 5.00)	0.01	-0.06 (-2.23, 2.12)	0.96	-2.90 (-5.00, -0.80)	0.01
Triglycerides (mmol/L)	0.11 (-0.26, 0.47)	0.56	-0.38 (-0.75, -0.01)	0.04	-0.49 (-0.84, -0.14)	0.01
No NAFLD						
Liver fat (%)	0.08 (-0.63, 0.79)	0.83	-0.99 (-1.60, -0.38)	0.002	-1.07 (-1.96, -0.18)	0.02
LDL cholesterol (mmol/L)	-0.10 (-0.38, 0.18)	0.47	-0.46 (-0.77, -0.15)	0.004	-0.36 (-0.69, -0.04)	0.03
Stratified by PNPLA3 (I148M) genotype						
PNPLA3 (CC)						
Liver fat (%)	0.48 (-0.34, 1.29)	0.25	-1.88 (-3.78, 0.02)	0.05	-2.35 (-4.37, -0.34)	0.02
HbA1c (mmol/L)	2.15 (0.39, 3.92)	0.01	-1.59 (-3.60, 0.43)	0.12	-3.74 (-5.74, -1.74)	0.0004
Total cholesterol (mmol/L)	0.21 (-0.17, 0.59)	0.27	-0.41 (-0.84, 0.01)	0.06	-0.62 (-1.04, -0.21)	0.004
LDL cholesterol (mmol/L)	0.18 (-0.13, 0.48)	0.26	-0.36 (-0.72, 0.00)	0.048	-0.54 (-0.87, -0.21)	0.002
Triglycerides (mmol/L)	0.06 (-0.21, 0.34)	0.64	-0.47 (-0.77, -0.17)	0.003	-0.54 (-0.84, -0.24)	0.001
ApoB (g/L)	0.03 (-0.05, 0.11)	0.42	-0.08 (-0.17, 0.01)	0.09	-0.11 (-0.20, -0.02)	0.02
PNPLA3 (CG/GG)						
HDL cholesterol (mmol/L)	-0.12 (-0.22, -0.02)	0.01	-0.10 (-0.20, 0.00)	0.04	0.02 (-0.08, 0.12)	0.69

[1]Intention-to-treat (ITT) point estimates and corresponding 95% confidence intervals (CI) are demonstrated for outcomes that were statistically significant in between-group comparisons of the general linear model (GLM)/Kruskal-Wallis (KW) test (**Table 3**). Estimates and two-sided p-values are derived from a GLM or the Willets residual method.

CRP, C-Reactive Protein; HbA1c, Hemoglobin A1c; HND, Healthy Nordic Diet; LCPUFA, Low Carbohydrate Polyunsaturated Fatty Acids; LDL, Low-Density Lipoprotein; M0, Baseline; M12, Month 12; UC, Usual Care.

Supplementary Table 8. Adverse events.

	LCPUFA (n=54)		HND (n=51)		UC (n=43)	
	M6-M0 (n=51)	M12-M6 (n=50)	M6-M0 (n=50)	M12-M6 (n=50)	M6-M0 (n=41)	M12-M6 (n=40)
Gastrointestinal issues (n (%))	5 (10)	5 (10)	5 (10)	2 (4)	4 (10)	1 (3)
Fatigue (n (%))	1 (2)	0 (0)	1 (2)	0 (0)	0 (0)	0 (0)
Headache (n (%))	0 (0)	0 (0)	1 (2)	1 (2)	0 (0)	1 (3)
Stress/anxiety (n (%))	0 (0)	0 (0)	0 (0)	0 (0)	0 (0)	0 (0)
Covid-19 (n (%))	1 (2)	3 (6)	1 (2)	1 (2)	1 (2)	2 (5)
Cold/fever (n (%))	3 (6)	1 (2)	1 (2)	0 (0)	1 (2)	3 (8)
Bodily pain (n (%))	4 (8)	1 (2)	6 (12)	2 (4)	6 (15)	3 (8)
Appetite loss (n (%))	0 (0)	0 (0)	0 (0)	0 (0)	0 (0)	0 (0)
Other (n (%))	6 (12)	4 (8)	3 (6)	3 (6)	9 (22)	7 (18)
Serious adverse events (n (%))	0 (0)	2 (4)	1 (2)	0 (0)	0 (0)	1 (3)
Total number adverse events (n)	20	16	19	9	21	18

LCPUFA, Low Carbohydrate Polyunsaturated Fatty Acids; HND, Healthy Nordic Diet; UC, Usual Care.

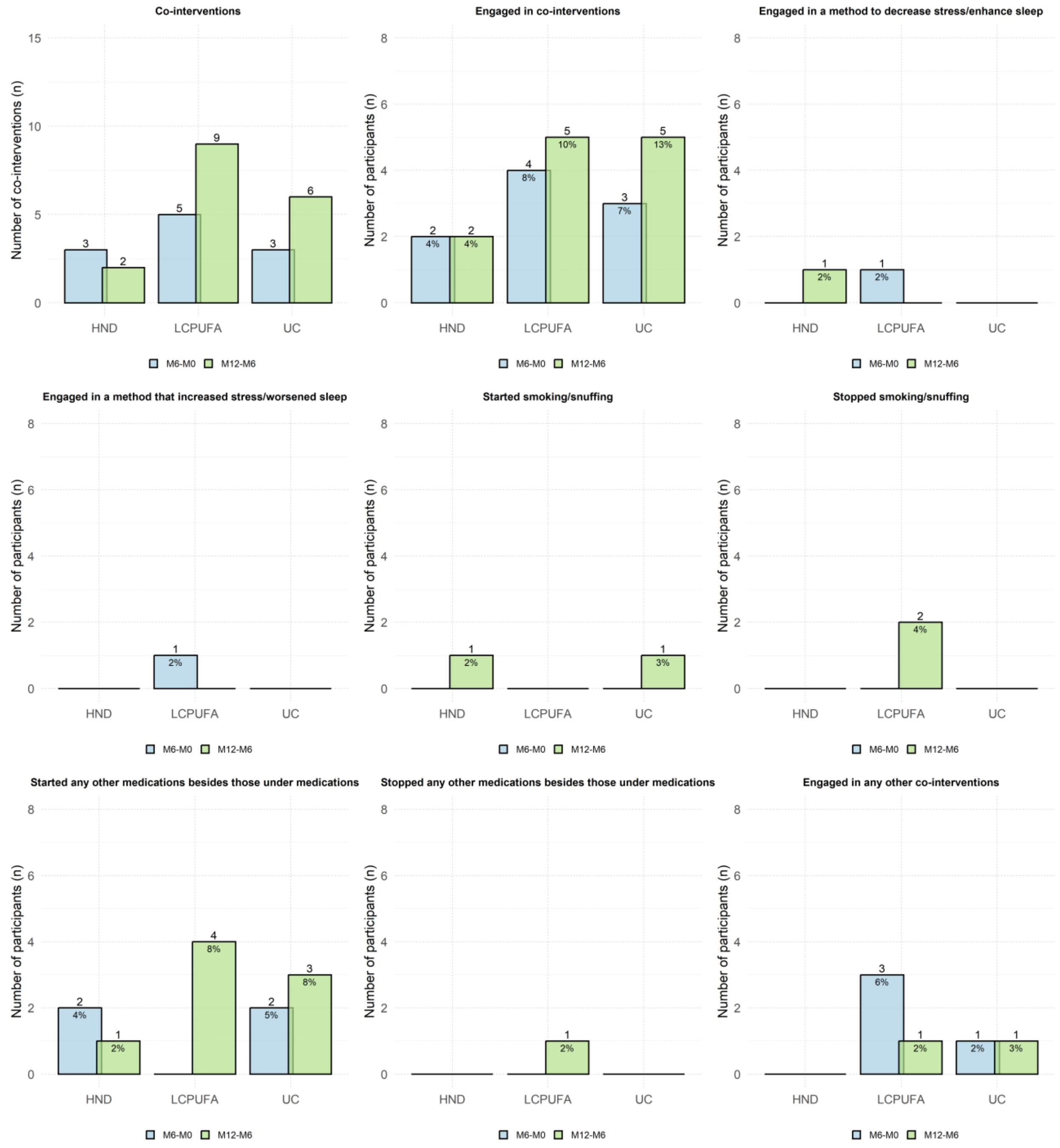

Supplementary Figure 2. Co-interventions other than medications, supplements, alcohol and physical activity between month 6 (M6) and baseline (M0) and between month 12 (M12) and M6. Data are presented as counts (n) on top of each bar and percentages (%) within each bar. Blue colored bars represent M6-M0 and green colored bars represent M12-M6. n (HND) = 50; n (LCPUFA) = 50-51 (for M12-M0 and M6-M0 respectively); n (UC) = 40-41 (for M12-M0 and M6-M0 respectively). HND, Healthy Nordic Diet; LCPUFA, Low Carbohydrate Polyunsaturated Fatty Acids; M, Month; UC, Usual Care. Source data are provided as a Source Data file.

Supplementary Table 9. Co-interventions from alcohol[1].

	LCPUFA (n=54)		HND (n=51)		UC (n=43)	
	M6-M0 (n=50-51)	M12-M6 (n=48-50)	M6-M0 (n=48-50)	M12-M6 (n=50)	M6-M0 (n=40)	M12-M6 (n=38-39)
PEth (µmol/L)	0.0 (0.0)	0.0 (0.0)	0.0 (0.0)	0.0 (0.0)	0.0 (0.0)	0.0 (0.0)
Alcohol (g/d)	0.0 (10.8)	0.0 (6.8)	0.0 (8.7)	0.0 (7.8)	0.0 (4.2)	0.0 (1.7)

[1]Data are presented as medians (interquartile range (IQR)).

Alcohol (g/d) is self-reported from 4 day weighed food records whereas concentration of PEth is measured from fasting blood samples.

HND, Healthy Nordic Diet; LCPUFA, Low Carbohydrate Polyunsaturated Fatty Acids; M, Month; PEth, Phosphatidylethanol; UC, Usual Care.

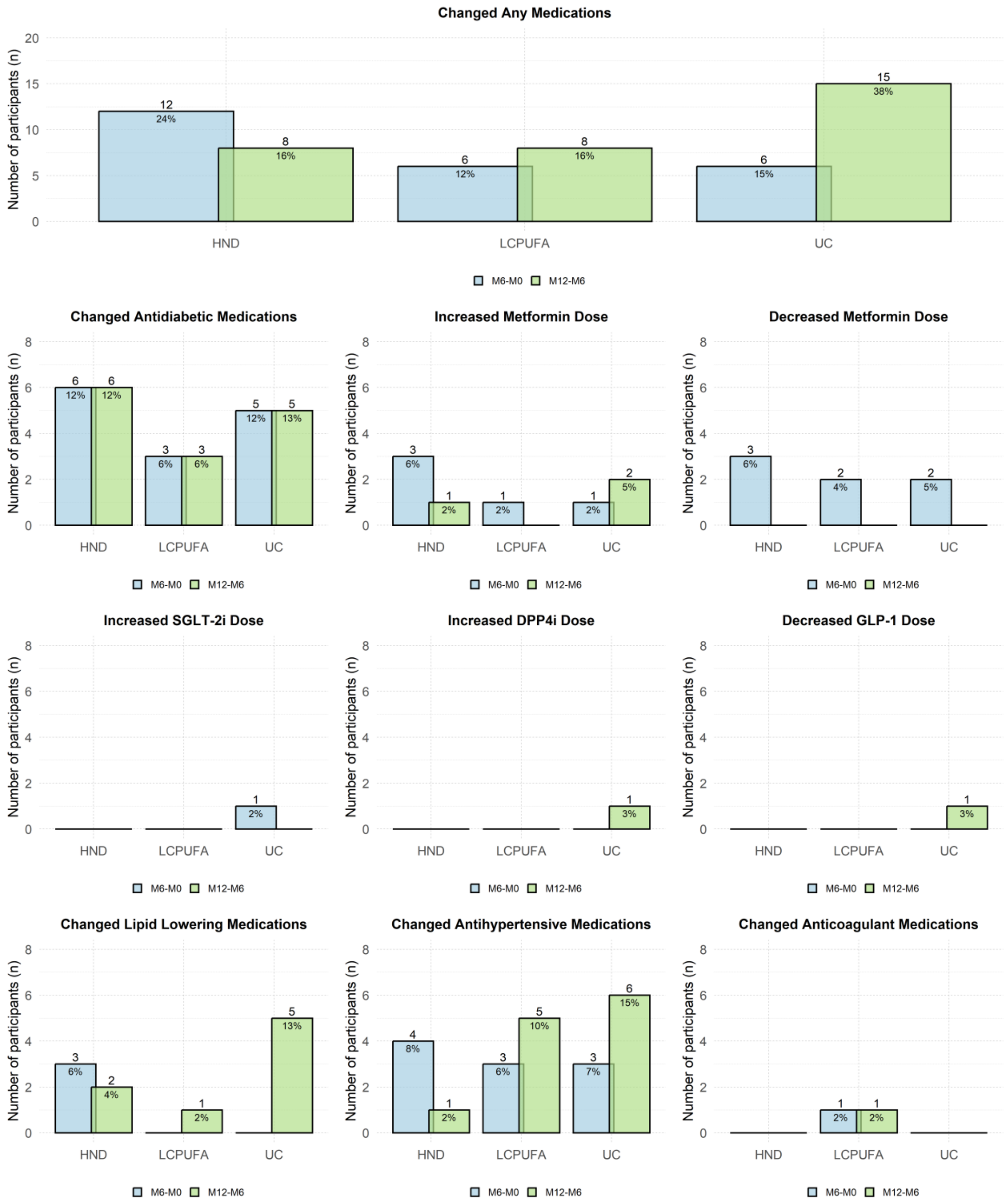

Supplementary Figure 3. Changes in antidiabetic-, antihypertensive- and lipid lowering medications between month 6 (M6) and baseline (M0) and between month 12 (M12) and M6. No participant decreased their dose of SGLT2i nor changed their use of sulfonylureas during the study period. Data are presented as counts (n) on top of each bar and percentages (%) within each bar. Blue colored bars represent M6-M0 and green colored bars represent M12-M6. n (HND) = 50; n (LCPUFA) = 50-51 (for M12-M0 and M6-M0 respectively); n (UC) = 40-41 (for M12-M0 and M6-M0 respectively). HND, Healthy Nordic Diet; LCPUFA, Low Carbohydrate Polyunsaturated Fatty Acids; UC, Usual Care. Source data are provided as a Source Data file.

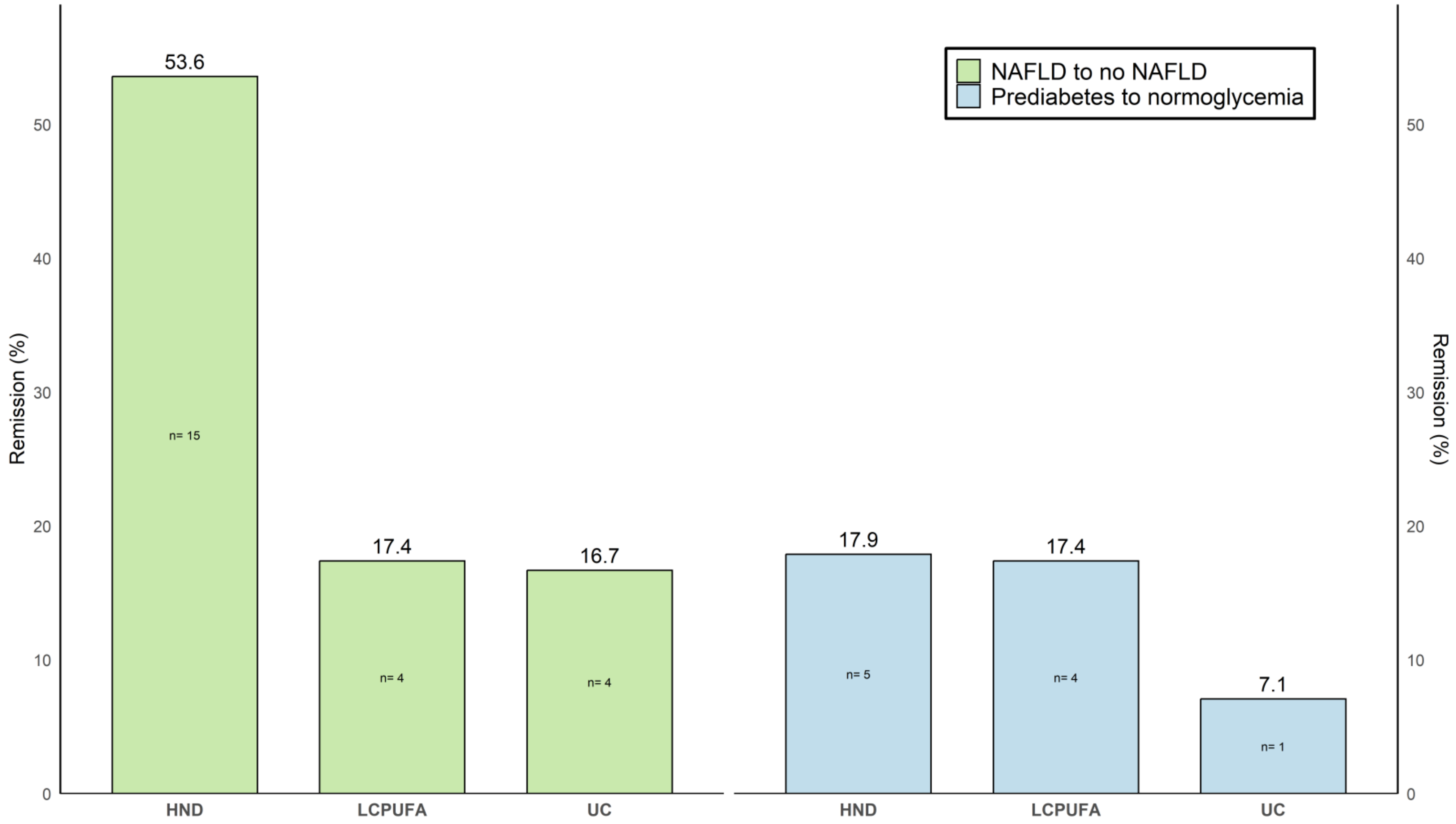

Supplementary Figure 4. Relative and absolute remission rates of prediabetes and NAFLD. Relative numbers are highlighted on top of each bar and absolute values are highlighted within each bar. Descriptive data is based on those with complete data on fasting plasma glucose and HbA1c values at baseline and follow-up (prediabetes) and liver fat at baseline and follow-up (NAFLD).

Green colored bars represent NAFLD to no NAFLD and blue colored bars represent prediabetes to normoglycemia.

HND, Healthy Nordic Diet; LCPUFA, Low Carbohydrate Polyunsaturated Fatty Acids; NAFLD, Non-Alcoholic Fatty Liver Disease; UC, Usual Care.

Source data are provided as a Source Data file.

Supplementary Table 10. PNPLA3 I148M genotype distributions among NAFLD subgroups.

	No NAFLD	NAFLD
PNPLA3 I148M CC n (%)	36 (64)	47 (55)
PNPLA3 I148M CG/GG n (%)	20 (36)	39 (45)

NAFLD, Non-Alcoholic Fatty Liver Disease; PNPLA3, Patatin-Like Phospholipase domain-containing protein 3.

Causal mediation analysis (CMA)

The total causal effect (TE) of an exposure on an outcome is from a counterfactual approach to causality decomposed into the natural indirect effect (NIE) and the natural direct effect (NDE). These causal estimands have been defined before [1]. The indirect effect is transmitted via a mediator on the outcome. In our study, the exposure is diet group, the mediator is weight change from month 0 to month 12 and the outcome is liver fat change from month 0 to month 12. Assuming no mediator-outcome confounding (assumption 1) and no exposure-induced mediator-outcome confounders (assumption 2), the above causal quantities can be estimated, allowing for an interaction between the exposure and the mediator. We furthermore assumed that weight change occurred before any changes in liver fat, although both outcomes were assessed at month 12. Although data on weight were available at month 6, this time point was deemed to be too far apart from the outcome at month 12 to play a clinically meaningful part in the mediated effect. A sensitivity analysis specifying the mediator at month 6 was however performed. Proportion mediated (PM) through weight change can be estimated by dividing the NIE by the TE. Confidence intervals around the PM-estimate are then calculated. The PM was the measure of interest for this CMA. Since treatment allocation was randomized, exposure-mediator (assumption 3) and exposure-outcome confounders (assumption 4) can be assumed to be absent. Identified mediator-outcome confounders (MOC) to adjust for were: baseline sex, age, T2D status, number of diabetes medications, education, weight, and liver fat at baseline. A simplified directed acyclic graph (DAG) depicting our causal assumptions is provided below [2].

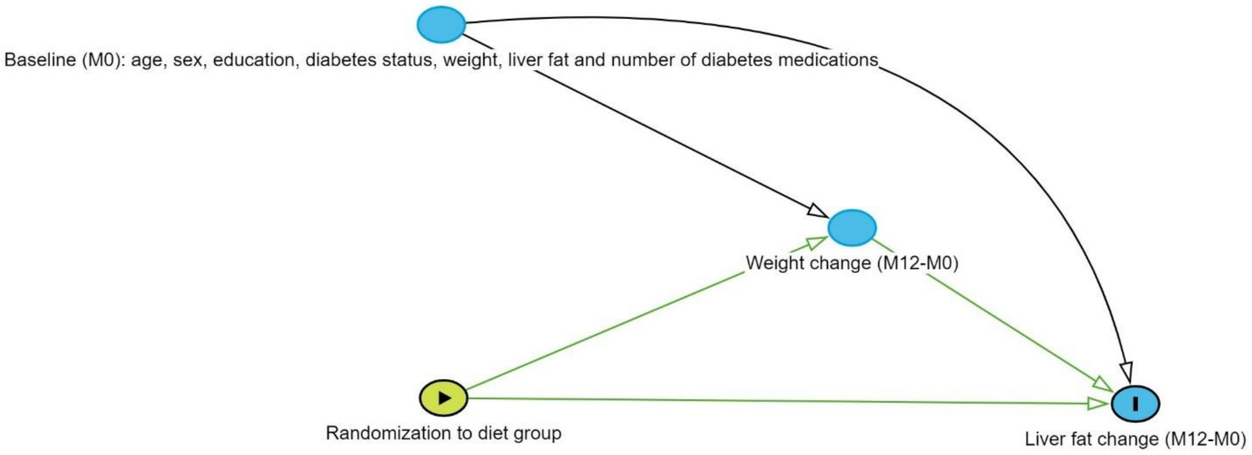

Supplementary Figure 5. A directed acyclic graph (DAG) depicting our causal assumptions of included variables. To estimate the proportion (PM) of the effect of diet on liver fat change (M12-M0), mediated through weight change (M12-M0), a minimal sufficient adjustment set of weight change and all mediator-outcome confounders (MOC); baseline age, sex, education, diabetes status, weight, liver fat and number of diabetes medication, was identified. MOC were identified from the background literature combined with subject-matter expertise. No arrows are directed to Randomization to diet group as diet allocation was randomized, hence exchangeability holds.

The counterfactual approach to mediation differs from a more traditional approach (e.g., the difference method) whereby two regression models are specified: one full model with the mediator and one partial model without the mediator. The direct effect (DE) is retrieved from the

exposure coefficient in the full model whereas the indirect effect (IE) is retrieved by calculating the difference between the exposure coefficients in the partial model and the full model. Importantly however, the direct and indirect effects from the difference method do not necessarily estimate causal quantities such as the NDE or NIE, except in cases when linear regression models are used for the mediator and outcome and there is no exposure-mediator interaction present. In addition, the traditional approach to mediation analysis usually does not include mediator-outcome confounders. One could also argue that the no exposure-mediator interaction assumption is less likely to hold for diet and weight change. Estimated DE and NIE may therefore be biased in the presence of MOC and interactions. To be able to compare estimates from our CMA against more traditional methods, the difference method was used to estimate PM by weight change, with and without the inclusion of MOC, but without an exposure-mediator interaction.

We used the regmedint package in R to estimate PM with corresponding 95% CI. In contrast to the primary analyses, liver fat was log-transformed before running this analysis. This was due to limitations in the R-package of handling non-parametric models. Missing values were imputed using MICE (mice), as described in the main text. An interaction term between diet and the mediator was specified and both the outcome model as well as the mediator model were specified as linear models. Although not the primary estimand of interest, we furthermore calculated the mediational E-value for the NIE (which is part of the PM-estimate) to examine the robustness to any unmeasured mediator-outcome confounding for the PM estimates that were statistically significant between groups [3]. The mediational E-value is an estimate of the minimum strength of association on the risk ratio scale that an unmeasured confounder must have with both the mediator and the outcome to fully explain away the NIE. A small E-value indicates that little unmeasured confounding is needed and vice versa for a big E-value.

Results and discussion of the CMA

Results from the CMA is presented in Supplementary Figure 6. PM by weight change was 74 (95% CI: -6, 153) % for LCPUFA vs HND, 21 (-38, 80) % for LCPUFA vs UC and 56 (5, 107) % for HND vs UC. PM estimates differed to a relatively minor degree between the different modelling approaches to mediation. The mediational E-value for the NIE of the comparison between the HND and UC was 2 for the point estimate and 1.25 for the upper 95% CI. Sensitivity analyses using weight at month 6 for the comparison between the HND and UC showed similar estimates as the primary analysis: 46 (95% CI: 6, 86) %. Although accompanied by wide CI, findings from this post-hoc analysis indicate that weight change is a significant contributor to the effects of diet on liver fat for the comparison between the HND and the other diets, but not so much for the comparison between the LCPUFA group and UC. Interestingly, the PM estimate for the comparison between the HND and UC indicate that 44 % of the effect on liver fat was not mediated through weight change.

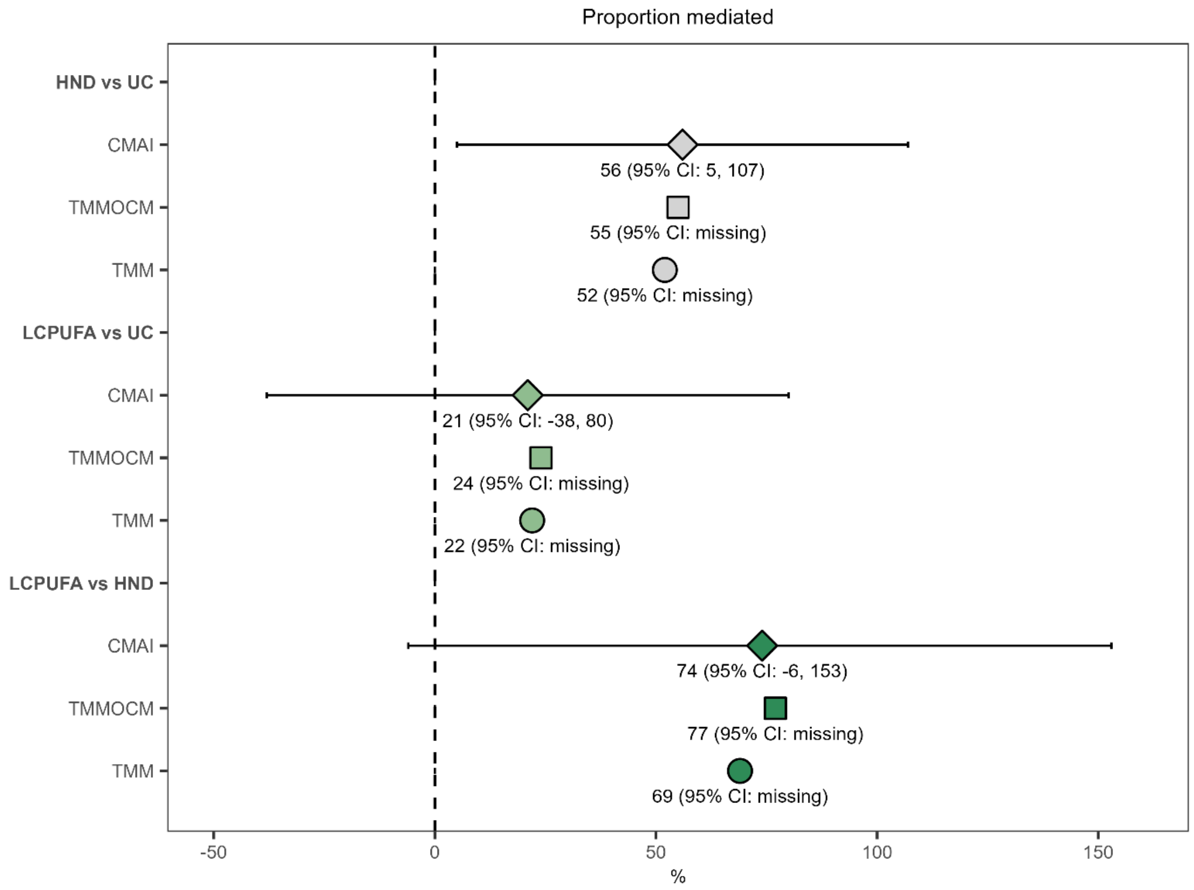

Supplementary Figure 6. Proportion mediated (PM) by weight change on the effect of diet on liver fat changes. Data are presented as % with corresponding 95% confidence intervals (CI) for the causal mediation analysis (CMA) but not for the traditional approaches (TMM and TMMOCM) in n=148 individuals. Causal mediation analyses were conducted using the regmedint package in R to estimate PM with corresponding 95% CI. In contrast to the primary analyses, liver fat was log-transformed before running this analysis. This was due to limitations in the R-package of handling non-parametric models. Missing values were imputed using MICE (mice in R), as described in the main text. An interaction term between diet and the mediator was specified and both the outcome model as well as the mediator model were specified as linear models.

CMAI, Causal Mediation Analysis with Interactions; HND, Healthy Nordic Diet; LCPUFA, Low Carbohydrate Polyunsaturated Fatty Acids; TMM, Traditional Model with Mediator; TMMOCM, Traditional Model with Mediator-Outcome Confounders and Mediator; UC, Usual Care.

Point estimates with grey color refer to the comparison between HND vs UC; light green LCPUFA vs UC; dark green LCPUFA vs HND.

Source data are provided as a Source Data file.

n (LCPUFA) = 54; n (HND) = 51; n (UC) = 43.

References

1. Li, Y., Yoshida, K., Kaufman, J. S. & Mathur, M. B. A brief primer on conducting regression-based causal mediation analysis. *Psychol. Trauma Theory, Res. Pract. Policy* **15**, 930–938 (2023).
2. Greenland, S., Pearl, J. & Robins, J. M. Causal diagrams for epidemiologic research. *Epidemiology* **10**, 37–48 (1999).
3. Smith, L. H. & VanderWeele, T. J. Mediational E-values. *Epidemiology* **30**, 835–837 (2019).

NAFLDiet study protocol

Principal investigator:	Ulf Risérus, professor<br>Uppsala University, Uppsala, Sweden
ClinicalTrials.gov identifier:	NCT04527965
Authors:	Fredrik Rosqvist, Associate professor<br>Uppsala University, Uppsala, Sweden
	Michael Fridén, PhD student<br>Uppsala University, Uppsala, Sweden
Biostatistician:	Lars Berglund, Associate professor<br>Uppsala University, Uppsala, Sweden

Start date:	2020-08-11
End date:	2022-12-20

Abbreviations

ApoA1	Apolipoprotein A1
ApoB	Apolipoprotein B
BIA	Bioelectrical impedance analysis
BMI	Body mass index
CRP	C-reactive protein
CVD	Cardiovascular disease
DNL	De-novo lipogenesis
FMD	Flow-mediated dilation
GC	Gas chromatography
GLM	General Linear Model
HbA1c	Glycated hemoglobin
HDL	High-density lipoprotein
HOMA-IR	Homeostatic Model Assessment for Insulin Resistance
IQR	Interquartile range
ITT	Intention to treat
LDL	Low-density lipoprotein
MRI	Magnetic resonance imaging
NAFLD	Non-alcoholic fatty liver disease
OGTT	Oral glucose tolerance test
PNPLA3	Patatin-like phospholipase domain protein 3
PP	Per protocol
PUFA	Polyunsaturated fatty acid(s)
PWV	Pulse-wave velocity
SCD-1	Stearoyl coenzyme-A desaturase 1
SD	Standard deviation
T2D	Type 2 diabetes
UPLC-MS/MS	Ultra-high performance liquid chromatography coupled to tandem mass spectrometry

Table of contents

1. Introduction……………………………………………………………………………..4
2. Study design…………………………………………………………………………...4-6
    2.1. Sample size calculation……………………………………………….................6
3. Aims and objectives……………………………………………………….................6
4. Outcomes………………………………………………………………………….6-10
    4.1. Primary outcome……………………………………………………………………6
    4.2. Secondary outcomes………………………………………………………...6-9
    4.3. Exploratory outcomes……………………………………………………………9-10
    4.4. Safety outcomes…………………………………………………………………....10
    4.5. Co-interventions..10
5. Populations and subgroups to be analyzed…………………………………………10-11
    5.1. Populations………………………………………………………………………...10
    5.2. Subgroups………………………………………...……………………….....10-11
6. Analyses………………………………………………………………………………11-12
    6.1. Primary outcome……………………………………………………………11-12
    6.2. Secondary and exploratory outcomes……………………………………………...12
    6.3. Other statistical analyses that will be conducted…………………………………..12
7. Missing data………………………………………………………………………….....12
8. Deviations from the study protocol……………………...…………………………...13
9. Summary of the study protocol………………………………...………………...….14
10. References………………………………………………………………………….......15

1. Introduction

Randomized controlled studies investigating the impact of replacing dietary carbohydrates with polyunsaturated fat (PUFA) on liver fat content and cardiometabolic risk in individuals with prediabetes and T2D are lacking. Also, the effects of a Healthy Nordic Diet on liver fat content and glycemic control have not be investigated. This study therefore aims to:

- Investigate the effects of the diets on liver fat content (primary aim)
- Investigate the effects of the diets on pancreatic fat, visceral fat, lean tissue, glycemic and lipid control
- Investigate the effects of the diets on plasma markers of de novo lipogenesis (DNL) and desaturation (i.e. stearoyl-Coenzyme desaturase 1, SCD-1) as well as on hepatic DNL using MRI spectroscopy
- Investigate gene-diet interactions, especially if common gene variants (e.g. in PNPLA3) known to increase liver fat and dyslipidemia, may modify the dietary effects.
- Perform lipidomic analyses to identify potential mechanistic pathways that may associate with diet-induced changes in liver fat, pancreatic fat, visceral fat, insulin sensitivity, dyslipidemia or DNL

Our hypothesis is that a customized diet will effectively reduce liver fat through suppression of hepatic DNL and SCD-1 activity, and thereby improve atherogenic dyslipidemia, insulin resistance and hyperglycemia in individuals with prediabetes and T2D.

2. Study design

Study Type: Interventional (Single-center Clinical Trial)

Estimated Enrollment: 150 participants

Allocation: Randomized

Intervention Model: Parallel assignment

Intervention Model Description: Parallel assignment

Masking: Double (Care Provider, Outcomes Assessors)

Primary Purpose: Treatment

Hypothesis Testing Framework: Superiority Trial

Eligibility criteria are presented below:

Inclusion criteria

- Men and women
- 30-75 years
- BMI 25-40
- T2D (duration ≤10 years, no insulin treatment) or prediabetes (ADA definition 2019) without diagnosed cardiovascular disease (CVD) during the last 2 years (e.g. myocardial infarction, stroke or angina pectoris)

Exclusion criteria

- BMI >40
- Alcohol intake >20 g/day
- Unwillingness to follow a new prescribed diet for 1 year
- Diet-induced weight loss (≥10%) the preceding 3 months of screening
- Malignant disease
- Severe kidney and liver disease
- Heart failure or other severe CVD
- Claustrophobia or metal parts in the body (MRI)

Subjects enrolled in this study will be allocated to three different diet groups in a 1:1:1 allocation ratio using stratified randomization with type-2 diabetes status (yes/no) and gender (male/female) as stratifying factors. Subjects will be followed prospectively for 12 months.

The three diet-groups are:

Experimental: Customized diet to reduce liver fat
Ad libitum diet high in plant-derived PUFA and lower in carbohydrates

Experimental: Healthy Nordic diet
Ad libitum diet, based on Nordic foods, higher in carbohydrates (high fiber/low GI) and lower in fat but rich in monounsaturated fatty acids (MUFA) and PUFA

Active Comparator: Control
Ad libitum diet in accordance with the Nordic Nutrition Recommendations

Further details of each diet can be found in the study protocol registered at ClinicalTrials.gov (NCT04527965).

2.1. Sample size calculation

The sample size calculation was based on Lehr´s formula for comparison between two groups, assuming equal treatment effects for the two experimental diets. The standard deviation (SD) of the change in liver fat was 1.95 and 2.42 in two of our previous trials (1,2). As the population in the NAFLDiet trial will differ (e.g. higher prevalence of type 2 diabetes) compared to these other trials, we assume a SD of 3 for the current calculation. Given that 5% liver fat is the cut-off for NAFLD, we considered a 2% difference between groups as clinically relevant. To detect a difference of 2%-points in liver fat between the control group and one experimental group, n=36 individuals per group are needed with β=0.80 and α=0.05. To allow for 25% drop-out, n=50 individuals per group will be included. Thus, a total of n=150 individuals will be randomized.

3. Aims and objectives

The overall aim of this study is to investigate the long-term impact of a customized diet aimed at reducing liver fat specifically and a healthy Nordic diet on ectopic fat (liver, pancreatic and visceral) and cardiometabolic risk in individuals with prediabetes and type 2 diabetes.

4. Outcomes

4.1. Primary outcome

- Between-group changes in liver fat content between baseline and month 12

  [Time Frame: 12 months] [Unit: %] [Variable: Continuous]

  Assessed by magnetic resonance imaging (MRI)

4.2. Secondary outcomes

- Between-group changes in visceral adipose tissue mass between baseline and month 12

  [Time Frame: 12 months] [Unit: L] [Variable: Continuous]

  Assessed by magnetic resonance imaging (MRI)

- Between-group changes in lean tissue mass between baseline and month 12

  [Time Frame: 12 months] [Unit: L] [Variable: Continuous]

  Assessed by magnetic resonance imaging (MRI)

- Between-group changes in total body fat mass between baseline and month 12

  [Time Frame: 12 months] [Unit: L] [Variable: Continuous]

  Assessed by magnetic resonance imaging (MRI)

- Between-group changes in body weight between baseline and month 12

  [Time Frame: 12 months] [Unit: kg] [Variable: Continuous]

  Assessed by using a Tanita bioelectrical impedance analysis (BIA) scale

- Between-group changes in glycated hemoglobin (HbA1c) between baseline and month 12

  [Time Frame: 12 months] [Unit: mmol/mol] [Variable: Continuous]

  Assessed by routine clinical chemistry

- Between-group changes in Homeostatic Model Assessment for Insulin Resistance (HOMA-IR) between baseline and month 12

  [Time Frame: 12 months] [Unit: none] [Variable: Continuous]

  Calculated by multiplying fasting plasma glucose (mmol/L) with fasting serum insulin (mU/L) and dividing the product by 22.5.

  Assessed by routine clinical chemistry

- Between-group changes in fasting plasma glucose between baseline and month 12

  [Time Frame: 12 months] [Unit: mmol/L] [Variable: Continuous]

  Assessed by routine clinical chemistry

- Between-group changes in fasting serum insulin between baseline and month 12

  [Time Frame: 12 months] [Unit: mU/L] [Variable: Continuous]

Assessed by routine clinical chemistry

- Between-group changes in systolic blood pressure between baseline and month 12

  [Time Frame: 12 months] [Unit: mmHg] [Variable: Continuous]

  Assessed by using an automated blood pressure monitor

- Between-group changes in diastolic blood pressure between baseline and month 12

  [Time Frame: 12 months] [Unit: mmHg] [Variable: Continuous]

  Assessed by using an automated blood pressure monitor

- Between-group changes in plasma lipids (total cholesterol, LDL cholesterol, triglycerides, HDL cholesterol, apoB and apoA1) between baseline and month 12

  [Time Frame: 12 months] [Unit: mmol/L or g/L (apoB and apoA1)] [Variable: Continuous]

  Assessed by routine clinical chemistry

- Between-group changes in circulating inflammatory markers (CRP, Tumor Necrosis Factor Alpha-receptor 1 and 2, Interleukin-1 receptor antagonist, Fibroblast growth factor 21) between baseline and month 12

  [Time Frame: 12 months] [Unit: ng/l or mg/l (CRP)] [Variable: Continuous]

  Assessed by routine clinical chemistry and ELISA

- Between-group changes in pancreatic fat between baseline and month 12

  [Time Frame: 12 months] [Unit: %] [Variable: Continuous]

  Assessed by magnetic resonance imaging (MRI)

- Between-group changes in flow-mediated dilation (FMD) between baseline and month 12

  [Time Frame: 12 months] [Unit: %] [Variable: Continuous]

Assessed by ultrasound in approximately half of the study population (n=75)

- Between-group changes in pulse-wave velocity (PWV) between baseline and month 12

  [Time Frame: 12 months] [Unit: m/s] [Variable: Continuous]

  Assessed by ultrasound in approximately half of the study population (n=75)

- Between-group values in FMD and PWV at month 12

  [Unit: % and m/s] [Variable: Continuous]

  Assessed by ultrasound in the whole population (n=150)

- Between-group changes in liver fat, HbA1c, and blood lipids in prespecified subgroups of type-2 diabetes status (yes/no), gender (male/female), individuals with NAFLD/without NAFLD at baseline, individuals with the CC vs CG/GG I148M genotype in PNPLA3, and in individuals with low respectively high dietary compliance based on dietary and lipogenic biomarkers changes (e.g. linoleic acid, DHA and palmitoleic acid changes from baseline to 12 months) between baseline and month 12

  [Time Frame: 12 months]

  We will assess whether response to diets in liver fat, HbA1c and blood lipids differ between gender, high vs low compliers, genotype, individuals with prediabetes or diabetes or those who have NAFLD/do not have NAFLD at baseline

4.3. Exploratory outcomes

- Between-group changes in plasma and imaging-derived fatty acids and fatty acid ratios in the lipogenic pathway (i.e.16:1n-7, 18:1n-9, 16:0, 14:0, 18:0 and 16:1n-7/16:0, 16:0/18:2n-6, 16:1n-7/18:2n-6) between baseline and month 12

  [Time Frame: 12 months] [Unit: %] [Variable: Continuous]

  Fatty acids are measured as percentage change of all fatty acids, thus using one unit for the above five fatty acids. Assessed by gas chromatography (GC) and proton spectroscopy, and use of bioinformatic modelling (untargeted) to identify responders and non-responders in liver fat reduction and improvement in glycemic control and blood lipids (i.e. personalized medicine approach)

- Between-group changes in plasma lipids (ceramides) using a targeted lipidomic approach between baseline and month 12

  [Time Frame: 12 months] [Unit: absolute and relative amounts] [Variable: Continuous]

  Lipids are measured using ultra-high performance liquid chromatography coupled to tandem mass spectrometry (UPLC-MS/MS)

4.4. Safety outcomes

Adverse events
Adverse events are reported at each clinic visit (after 6 and 12 months follow-up) using administered questionnaires with open-ended questions. Adverse events are categorized into 10 subcategories (ranging from gastrointestinal issues to serious adverse events) and will be presented as both the total number of events in each diet group (n/%) and separately for each of the 10 events (n/%) at both 6 months- and 12 months follow up.

Interim analysis
No interim analysis will be performed.

4.5. Co-interventions

Information on co-interventions such as medication use and dietary supplements (among others) are reported at each clinic visit (after 6 and 12 months follow-up) using administered questionnaires with open-ended questions. Alcohol intake will be assessed using the concentration of the phosphatidylethanol biomarker in fasting blood samples and physical activity (step count) will be assessed using pedometers.

5. Populations and subgroups to be analyzed

5.1 Populations

Intention to treat (ITT): All subjects randomized at baseline will constitute the ITT-population and will be the primary study population for the analyses. Missing data will be imputed as described in section 7 in this document.

Per-protocol (PP): All subjects completing the study period with complete data on the outcome of interest will constitute the PP-population. The PP-population will be the secondary study population for the analyses.

5.2 Subgroups

Further statistical analyses of the primary and secondary outcomes (liver fat, HbA1c and blood lipids) will be performed in the following prespecified subgroups:

Type-2 diabetes status: Subjects will be divided into two subgroups based on type-2 diabetes status (yes/no) of which the other subgroup ("no") will constitute of subjects without type-2-diabetes but with prediabetes as defined by a fasting plasma glucose between 5.6-6.9 mmol/L and/or an HbA1c value between 39-47 mmol/mol. Type-2 diabetes diagnosis is verified by a clinician using medical records.

Gender: Subjects will be divided into two subgroups based on gender (male/female).

NAFLD status: Subjects will be divided into two subgroups based on NAFLD status (yes/no) in which NAFLD is defined as a liver fat content exceeding 5.56 % of liver tissue.

PNPLA3 genotype: Subjects will be divided into two subgroups based on the I148M variant of the PNPLA3 gene (CC vs CG/GG genotype).

Compliers: Subjects will be divided into two subgroups based on dietary adherence (high vs low compliers), assessed using both dietary and lipogenic biomarker changes (e.g. linoleic acid, DHA and palmitoleic acid) between month 0 and month 12.

All subgroup analyses will be conducted as described for the analyses of the full study population.

6. Analyses

Normally distributed variables will be presented as mean ± SD and skewed distributed variables will be presented as median (IQR). Continuous variables will be tested for normality using the Shapiro-Wilk W test and skewed data ($W<0.95$) will be logarithmically transformed or analyzed non-parametrically, where appropriate. Homogeneity of variances between groups will be examined visually. Categorical variables will be presented as counts or percentages (%). An α-value of 0.05 is set as the significance level.

6.1. Primary outcome

For the primary outcome a General Linear Model (GLM) will be applied using Δliver fat (%) between month 12 and baseline as the dependent variable, treatment group as factor and type-2 diabetes status (yes/no), gender (male/female) and baseline liver fat content (%) as included covariates. The GLM will be applied for the ITT-population (defined above) as well as for the PP-population (defined above). Independent samples post-hoc t-tests will follow if between-group statistical significance is observed.

A sensitivity analysis will be conducted for the primary outcome in both the ITT- and PP-population, with weight change included as an additional covariate in the GLM.

Treatment effects will be presented as mean or median differences (%) with 95% confidence intervals and with corresponding p-values.

When assumptions of the GLM are not satisfied, the Willett´s residual method will be applied, followed by Mann-Whitney U post-hoc tests.

6.2 Secondary and exploratory outcomes

For secondary and exploratory outcomes GLMs will be applied using Δvalues between baseline and month 12 for each continuous variable (see point 4.2 and 4.3) as dependent variables and type-2 diabetes status (yes/no), gender (male/female) and baseline outcome values as included covariates in the model. Outcome values from 6 months follow-up will be presented descriptively.

The GLM will be applied for the ITT-population (defined above) as well as for the PP-population (defined above) for HbA1c, LDL cholesterol, triglycerides and systolic- and diastolic blood pressure. For the remaining secondary and exploratory outcomes, a PP-population will be used. Independent samples post-hoc t-tests will follow if between-group statistical significance is observed.

Treatment effects will be presented as mean or median differences (%) with 95% confidence intervals and with corresponding p-values.

When assumptions of the GLM are not satisfied, the Willett´s residual method will be applied (3), followed by Mann-Whitney U post-hoc tests.

6.3. Other statistical analyses that will be conducted

Pearson correlations (or Spearman rank correlation for non-normally distributed variables) between changes in serum fatty acids (presented as % of all fatty acids in that lipid compartment) and changes in liver fat content (%) and changes in HbA1c (mmol/mol) will be performed.

Pearson correlations (or Spearman rank correlation for non-normally distributed variables) between changes in liver fat content (%) and changes in HbA1c (mmol/mol), HOMA-IR, fasting plasma glucose (mmol/L), fasting serum insulin (mU/L), pancreatic fat (%), LDL-cholesterol (mmol/L), HDL-cholesterol (mmol/L), triglycerides (mmol/L), apoB (g/L) and apoA1 (g/L) will be performed.

7. Missing data

Missing numerical and categorical data (including both dependent- and explanatory variables) will be imputed using the technique of multiple imputation by chained equations (MICE). Categorical data will be coded into dummy variables. Number of missing values for each variable of which have been imputed will be presented as n/% for each group separately and in total.

8. Deviations from the study protocol

10. Addition of FIB-4 as a secondary outcome. Added to ClinicalTrials.gov the 23rd of July 2022, but was not added to the original SAP (which was uploaded 12th of May 2021).

11. Addition of ALAT, ASAT and GGT as exploratory outcomes were added to the manuscript after completion of the study, but not to the SAP nor ClinicalTrials.gov.

12. Addition of dietary and biomarker variables (i.e. plasma alkylresorcinols and fatty acids) relevant for the evaluation of adherence as a secondary outcome. These outcomes were neither added to ClinicalTrials.gov nor to the original SAP.

13. The estimation of the ITT-effect was decided to apply for all primary and secondary outcomes, not just for HbA1c, LDL cholesterol, triglycerides and systolic- and diastolic blood pressure, as prespecified on page 12 in the original SAP. The main reason for this decision was that the ITT-effect was our primary effect of interest (prespecified in the original SAP), and hence should apply to all outcomes of interest. As the ITT-effect is an unbiased effect of treatment assignment at baseline, whereas the per-protocol effect may be biased in case of patient dropouts and non-adherence, the ITT-effect for all outcomes was deemed more reasonable.

14. Information on number of steps from pedometers are not presented in the main text due to logistic difficulties in providing participants with a fully functional one at each time point (i.e. some pedometers stopped working or were lost over the follow-up period).

15. Subgroup analyses based on compliers will not be conducted. The ITT-effect cannot be estimated if compliance to each diet is non-random and affected by pre- and post-baseline variables. In addition, compliance was not prespecified on an individual basis, hence the difficulties in estimating the non-naïve per-protocol effect.

16. Sensitivity analysis whereby weight change is included as a covariate in the model has been extended to form a causal mediation analysis to estimate the proportion mediated by weight change of the primary outcome (referred to as a post-hoc analysis in the study) (see causal mediation analysis section on page 23-25). The reason for the extension is that traditional methods such as including the mediator as a covariate in the regression model does not usually consider mediator-outcome confounders and interactions between the exposure and the mediator, potentially leading to biased estimates of the direct and indirect effects.

17. Additional sensitivity analyses not prespecified in the SAP have been performed (see statistical analysis section of the manuscript for detailed information).

18. Other secondary and exploratory outcomes and analyses will be incorporated in later publications.

9. Summary of the study protocol

	Screening	M0	M1-M5	M6	M7-M11	M12
Pre-screening phone call	X					
Screening visit[1]	X					
Screening blood samples[2]	X					
Randomization	X					
Fasting blood samples[3]		X		X		X
Patient information[4]		X		X		X
BIA		X		X		X
PWV and FMD		X				X
Fecal sample		X		X		X
Blood pressure		X		X		X
Return of WFD		X		X		X
Return of pedometers		X		X		X
Provision of key food items		X		X		X
MRI		X				X
Monthly visits to the clinic[5]			X			
Bimonthly visits to the clinic[6]					X	

BIA, Bioelectrical Impedance Analysis; FMD, Flow-Mediated Dilation; M, Month; MRI, Magnetic Resonance Imaging; PWV, Pulse-Wave Velocity; WFD, Weighed Food Diaries.

[1]Involved assessment of weight, BMI and blood pressure as well as a questionnaire on lifestyle habits, medications, current and previous diseases
[2]Included standard clinical biomarkers measured at Uppsala University Hospital
[3]Included standard clinical biomarkers measured at Uppsala University Hospital
[4]Included questionnaires on demographics (M0), lifestyle habits (M0-M12), use of medications (M0-M12), adverse events (M6-M12) as well as questions on how participants perceived the diets (M12)
[5]A short meeting with the study coordinator, assessment of weight and provision of key food items
[6]A short meeting with the study coordinator, assessment of weight and provision of key food items

(yellow)	= Study coordinator
(green)	= Research nurse
(orange)	= Radiologist
(blue)	= Researcher not in contact with the participants

10. References

1. Bjermo H, Iggman D, Kullberg J, Dahlman I, Johansson L, Persson L, Berglund J, Pulkki K, Basu S, Uusitupa M, Rudling M, Arner P, Cederholm T, Ahlström H, Risérus U. Effects of n-6 polyunsaturated fat compared with saturated fat on liver fat, lipoproteins and inflammation in abdominal obesity: a randomized controlled trial. Am J Clin Nutr 2012;95:1003-12.

2. Rosqvist F, Kullberg J, Ståhlman M, Cedernaes J, Heurling K, Johansson H-E, et al. Overeating Saturated Fat Promotes Fatty Liver and Ceramides Compared With Polyunsaturated Fat: A Randomized Trial. J Clin Endocrinol Metab 2019;104(12):6207-19.

3. Willett W, Stampfer MJ. Total energy intake: implications for epidemiologic analyses. Am J Epidemiol 1986;124(1):17-27.

CONSORT 2010 checklist of information to include when reporting a randomised trial*

Section/Topic	Item No	Checklist item	Reported on page No
**Title and abstract**			
	1a	Identification as a randomised trial in the title	1
	1b	Structured summary of trial design, methods, results, and conclusions (for specific guidance see CONSORT for abstracts)	2,3
**Introduction**			
Background and objectives	2a	Scientific background and explanation of rationale	4,5
	2b	Specific objectives or hypotheses	4,5
**Methods**			
Trial design	3a	Description of trial design (such as parallel, factorial) including allocation ratio	19
	3b	Important changes to methods after trial commencement (such as eligibility criteria), with reasons	N/A
Participants	4a	Eligibility criteria for participants	19,20
	4b	Settings and locations where the data were collected	19
Interventions	5	The interventions for each group with sufficient details to allow replication, including how and when they were actually administered	20-23
Outcomes	6a	Completely defined pre-specified primary and secondary outcome measures, including how and when they were assessed	25,26
	6b	Any changes to trial outcomes after the trial commenced, with reasons	Suppl.
Sample size	7a	How sample size was determined	26,27
	7b	When applicable, explanation of any interim analyses and stopping guidelines	N/A
Randomisation:			
Sequence generation	8a	Method used to generate the random allocation sequence	20
	8b	Type of randomisation; details of any restriction (such as blocking and block size)	20

Allocation concealment mechanism	9	Mechanism used to implement the random allocation sequence (such as sequentially numbered containers), describing any steps taken to conceal the sequence until interventions were assigned	20
Implementation	10	Who generated the random allocation sequence, who enrolled participants, and who assigned participants to interventions	20
Blinding	11a	If done, who was blinded after assignment to interventions (for example, participants, care providers, those assessing outcomes) and how	20
	11b	If relevant, description of the similarity of interventions	N/A
Statistical methods	12a	Statistical methods used to compare groups for primary and secondary outcomes	26-29
	12b	Methods for additional analyses, such as subgroup analyses and adjusted analyses	28,29
**Results**			
Participant flow (a diagram is strongly recommended)	13a	For each group, the numbers of participants who were randomly assigned, received intended treatment, and were analysed for the primary outcome	Figure 1
	13b	For each group, losses and exclusions after randomisation, together with reasons	6, Figure 1
Recruitment	14a	Dates defining the periods of recruitment and follow-up	19
	14b	Why the trial ended or was stopped	N/A
Baseline data	15	A table showing baseline demographic and clinical characteristics for each group	Table 1
Numbers analysed	16	For each group, number of participants (denominator) included in each analysis and whether the analysis was by original assigned groups	6, Table 2-4
Outcomes and estimation	17a	For each primary and secondary outcome, results for each group, and the estimated effect size and its precision (such as 95% confidence interval)	7-9, Table 2-3, Figure 4
	17b	For binary outcomes, presentation of both absolute and relative effect sizes is recommended	N/A
Ancillary analyses	18	Results of any other analyses performed, including subgroup analyses and adjusted analyses, distinguishing pre-specified from exploratory	9-11, Figure 2-4, Table 2,4, Suppl.
Harms	19	All important harms or unintended effects in each group (for specific guidance see CONSORT for harms)	11, Suppl.

**Discussion**			
Limitations	20	Trial limitations, addressing sources of potential bias, imprecision, and, if relevant, multiplicity of analyses	16-18
Generalisability	21	Generalisability (external validity, applicability) of the trial findings	17
Interpretation	22	Interpretation consistent with results, balancing benefits and harms, and considering other relevant evidence	12-18
**Other information**			
Registration	23	Registration number and name of trial registry	19
Protocol	24	Where the full trial protocol can be accessed, if available	ClinicalTrials.gov Identifier: NCT04527965
Funding	25	Sources of funding and other support (such as supply of drugs), role of funders	38

*We strongly recommend reading this statement in conjunction with the CONSORT 2010 Explanation and Elaboration for important clarifications on all the items. If relevant, we also recommend reading CONSORT extensions for cluster randomised trials, non-inferiority and equivalence trials, non-pharmacological treatments, herbal interventions, and pragmatic trials. Additional extensions are forthcoming: for those and for up to date references relevant to this checklist, see www.consort-statement.org.
