# Supplementary material for: Effects of an anti-lipogenic low-carbohydrate high polyunsaturated fat diet or a healthy Nordic diet versus usual care on liver fat and cardiometabolic disorders in type 2 diabetes or prediabetes: a randomized controlled trial (NAFLDiet)
Source: Nat Commun. 2025 Dec 13;16:11130. doi: 10.1038/s41467-025-65613-2 (PMC12705689; doi:10.1038/s41467-025-65613-2)
Supplement: Supplementary file 2 — Reporting summary [file 41467_2025_65613_MOESM2_ESM.pdf]

nature portfolio

Corresponding author(s): Ulf Risérus
Last updated by author(s): Aug 30, 2025

Reporting Summary

Nature Portfolio wishes to improve the reproducibility of the work that we publish. This form provides structure for consistency and transparency in reporting. For further information on Nature Portfolio policies, see our Editorial Policies and the Editorial Policy Checklist.

Statistics

For all statistical analyses, confirm that the following items are present in the figure legend, table legend, main text, or Methods section.

n/a	Confirmed	
☐	☒	The exact sample size (*n*) for each experimental group/condition, given as a discrete number and unit of measurement
☐	☒	A statement on whether measurements were taken from distinct samples or whether the same sample was measured repeatedly
☐	☒	The statistical test(s) used AND whether they are one- or two-sided<br>*Only common tests should be described solely by name; describe more complex techniques in the Methods section.*
☐	☒	A description of all covariates tested
☐	☒	A description of any assumptions or corrections, such as tests of normality and adjustment for multiple comparisons
☐	☒	A full description of the statistical parameters including central tendency (e.g. means) or other basic estimates (e.g. regression coefficient) AND variation (e.g. standard deviation) or associated estimates of uncertainty (e.g. confidence intervals)
☐	☒	For null hypothesis testing, the test statistic (e.g. *F*, *t*, *r*) with confidence intervals, effect sizes, degrees of freedom and *P* value noted<br>*Give P values as exact values whenever suitable.*
☒	☐	For Bayesian analysis, information on the choice of priors and Markov chain Monte Carlo settings
☒	☐	For hierarchical and complex designs, identification of the appropriate level for tests and full reporting of outcomes
☒	☐	Estimates of effect sizes (e.g. Cohen's *d*, Pearson's *r*), indicating how they were calculated

Our web collection on statistics for biologists contains articles on many of the points above.

Software and code

Policy information about availability of computer code

Data collection	Data obtained from the participants were collected using case report forms in paper format and then transferred manually to excel-sheets. Data obtained from collaborators, such as MRI-data, genetics and alkylresorcinols were retrieved in processed excel-sheets.
Data analysis	R (R Core Team, Vienna, Austria) version 4.2.3 and IBM SPSS Statistics version 28.0.1.0 (142) were used to analyze the data. R-packages used to analyze the data were "mice", "bootimpute", "regmedint", among other commonly used R-packages. No custom code has been developed.

For manuscripts utilizing custom algorithms or software that are central to the research but not yet described in published literature, software must be made available to editors and reviewers. We strongly encourage code deposition in a community repository (e.g. GitHub). See the Nature Portfolio guidelines for submitting code & software for further information.

Data

Policy information about availability of data

All manuscripts must include a data availability statement. This statement should provide the following information, where applicable:

- Accession codes, unique identifiers, or web links for publicly available datasets
- A description of any restrictions on data availability
- For clinical datasets or third party data, please ensure that the statement adheres to our policy

All relevant data supporting the findings of this study are available within the main manuscript, the Supplementary Material, or as Source Data. Because the study includes sensitive patient data, the pseudonymized individual-level data from the NAFLDiet study cannot be made publicly available. Access may be granted upon

reasonable request to the corresponding author, but requires a clear project plan and prior approval from the Swedish Ethical Review Authority. The data will be shared exclusively for research purposes. Inquiries will be answered within approximately one month. The study protocol, statistical analysis plan (SAP), and CONSORT checklist are available in the Supplementary Material. Source data are provided with this paper.

Research involving human participants, their data, or biological material

Policy information about studies with human participants or human data. See also policy information about sex, gender (identity/presentation), and sexual orientation and race, ethnicity and racism.

Reporting on sex and gender	Both men and women (biological attribute) were recruited. Sex-specific subgroup analyses were conducted.
Reporting on race, ethnicity, or other socially relevant groupings	No reporting on race, ethnicity or other socially relevant groupings is available. Level of self-reported education (categorized into 9 years, 10-12 years or > 12 years of education) status was collected at baseline.
Population characteristics	Sex, age, BMI, current smoking, education, prevalence of type-2 diabetes, alcohol consumption, standard clinical biomarkers such as plasma glucose, LDL-cholesterol and CRP as well as body composition measures such as weight, BMI, liver fat, medication use, allele frequencies of PNPLA3 I148M genotype and self-reported dietary data.<br><br>Study participants were 65 (10) years old at baseline, had a BMI of 30.1±3.6 kg/m2 (LCPUFA), 29.5±3.7 kg/m2 (HND), 30.3 ±3.2 kg/m2 (UC) and a distribution of women/men of 41/59 % (LCPUFA), 39/61 % (HND) and 37/63 % (UC). Median glucose levels were comparable between groups whereas median percentage of liver fat was 6.3 (6.9) % for LCPUFA, 6.4 (5.5) % for HND and 8.7 (10.8) % for UC.
Recruitment	Participants were predominantly recruited from a local diabetes register (ANDiU) and a large population-based cohort (EpiHealth), but also through web-based advertisement.<br><br>Results from this study may not necessarily be validly extended to other populations with other distributions of potential effect modifiers (such as T2D status, NAFLD status and PNPLA3 allele frequencies, as indicated in our study). In addition, the intention-to-treat estimate is dependent on study-specific adherence to the diets, and hence, any other adherence patterns may provide other ITT-estimates. However, as outlined in the discussion, adherence (using self-reported data and fatty acid biomarkers) was good on a group-level.
Ethics oversight	The Ethical Review Board of Sweden.

Note that full information on the approval of the study protocol must also be provided in the manuscript.

Field-specific reporting

Please select the one below that is the best fit for your research. If you are not sure, read the appropriate sections before making your selection.

☒ Life sciences ☐ Behavioural & social sciences ☐ Ecological, evolutionary & environmental sciences

For a reference copy of the document with all sections, see nature.com/documents/nr-reporting-summary-flat.pdf

Life sciences study design

All studies must disclose on these points even when the disclosure is negative.

Sample size	Sample size calculation was based on Lehr´s formula for the comparison between groups, assuming equal treatment effects for the two experimental diets. A sample size of n=37 in each group was estimated to detect a 2 (SD: ± 3) percentage unit difference in liver fat between the experimental groups and the UC group, with significance level (α) of 0.05 and power (1-β) of 0.80. To achieve the desired power for both the intention-to-treat and the per-protocol analyses, and allowing a 25% dropout rate, we included 50 participants in each group.
Data exclusions	n=2 individuals who dropped out of the study after randomization but before being informed of their respective diet were excluded from the ITT-population. Missing data for outcomes were imputed using multivariate imputation using chained equations (MICE).
Replication	Data analyses were checked using both R and SPSS, in close collaboration with a biostatistician (L.B). No other data replications were performed.
Randomization	Randomization (stratified by sex and T2D status) was performed by a researcher not involved in the execution of the study (i.e. no contact with participants and no involvement in primary or secondary outcome ascertainments), using a computerized random-number generator. Knowledge of the allocation sequence was restricted to this researcher.
Blinding	Personnel responsible for assessing study outcomes (including dietary adherence) as well as care providers were blinded to the assigned diets of the participants. Neither the participants nor the study coordinator (M.F) were blinded to the assigned diets. Participants did not know of their assigned diet until they had completed all measurements from the fasting study baseline visit.

Reporting for specific materials, systems and methods

We require information from authors about some types of materials, experimental systems and methods used in many studies. Here, indicate whether each material, system or method listed is relevant to your study. If you are not sure if a list item applies to your research, read the appropriate section before selecting a response.

Materials & experimental systems

n/a	Involved in the study
☒	☐ Antibodies
☒	☐ Eukaryotic cell lines
☒	☐ Palaeontology and archaeology
☒	☐ Animals and other organisms
☐	☒ Clinical data
☒	☐ Dual use research of concern
☒	☐ Plants

Methods

n/a	Involved in the study
☒	☐ ChIP-seq
☒	☐ Flow cytometry
☒	☐ MRI-based neuroimaging

Clinical data

Policy information about clinical studies
All manuscripts should comply with the ICMJE guidelines for publication of clinical research and a completed CONSORT checklist must be included with all submissions.

Clinical trial registration	NCT04527965
Study protocol	NCT04527965. The SAP is publically available at ClinicalTrials.gov under this identification number.
Data collection	The NAFLDiet study was a RCT including three diet groups. Data on primary and secondary outcomes were collected at the Uppsala University Hospital in Uppsala (Sweden) between August 2020 and December 2022. The first participant was recruited in August of 2020 and the last participant was recruited in December of 2021. Data on diet, clinical biomarkers, liver fat and other prespecified outcomes were collected from August 2020 to December 2022. Complete processed data sets on liver fat were retrieved in July of 2023. Data on alkylresorcinols were retrieved in June of 2024, genetics (PNPLA3) in August of 2023 and fatty acids in February of 2025.
Outcomes	Liver fat was the primary outcome of interest whereas other cardiometabolic markers associated with NAFLD, T2D or prediabetes (relevant for this study population) were determined to be secondary outcomes. Liver fat was measured using magnetic resonance imaging (MRI) whereas other secondary outcomes were measured using routine laboratory methods at Uppsala University Hospital., bioelectrical impedance analysis (BIA) for weight and an automated blood pressure monitor for systolic- and diastolic blood pressure.

Plants

Seed stocks	N/A
Novel plant genotypes	N/A
Authentication	N/A
